# A chelicerate Wnt gene expression atlas: novel insights into the complexity of arthropod Wnt-patterning

**DOI:** 10.1186/s13227-021-00182-1

**Published:** 2021-11-09

**Authors:** Ralf Janssen, Matthias Pechmann, Natascha Turetzek

**Affiliations:** 1 Department of Earth Sciences, Palaeobiology, Uppsala University, Villavägen 16, 75236 Uppsala, Sweden; 2 Department of Developmental Biology, Biocenter, Institute for Zoology, University of Cologne, Zuelpicher Str. 47b, 50674 Cologne, Germany; 3 Evolutionary Ecology, Faculty of Biology, Ludwig-Maximilians Universität München, Grosshaderner Strasse 2, 82152 Biozentrum, Germany

**Keywords:** Wnt, Mygalomorpha, Opiliones, Spiders, Appendage development, Arthropod evolution, Gene duplication

## Abstract

The Wnt genes represent a large family of secreted glycoprotein ligands that date back to early animal evolution. Multiple duplication events generated a set of 13 Wnt families of which 12 are preserved in protostomes. Embryonic Wnt expression patterns (Wnt-patterning) are complex, representing the plentitude of functions these genes play during development. Here, we comprehensively investigated the embryonic expression patterns of Wnt genes from three species of spiders covering both main groups of true spiders, Haplogynae and Entelegynae, a mygalomorph species (tarantula), as well as a distantly related chelicerate outgroup species, the harvestman *Phalangium opilio*. All spiders possess the same ten classes of Wnt genes, but retained partially different sets of duplicated Wnt genes after whole genome duplication, some of which representing impressive examples of sub- and neo-functionalization. The harvestman, however, possesses a more complete set of 11 Wnt genes but with no duplicates. Our comprehensive data-analysis suggests a high degree of complexity and evolutionary flexibility of Wnt-patterning likely providing a firm network of mutational protection. We discuss the new data on Wnt gene expression in terms of their potential function in segmentation, posterior elongation, and appendage development and critically review previous research on these topics. We conclude that earlier research may have suffered from the absence of comprehensive gene expression data leading to partial misconceptions about the roles of Wnt genes in development and evolution.

**Supplementary Information:**

The online version contains supplementary material available at 10.1186/s13227-021-00182-1.

## Introduction

Wnt genes are important for the regulation of many aspects of animal development (reviewed in [92]. They encode secreted glycoprotein ligands that bind to different families of transmembrane receptors such as Frizzled and LRP5/6 (reviewed in e.g., [51]. Binding of Wnt molecules to their dedicated receptors activates intracellular signaling cascades that regulate target gene transcription (reviewed in e.g., [69, 88, 78]).

The last common ancestor of arthropods possessed 12 Wnt genes. However, loss of Wnt genes is common among arthropods [21, 30, 39], which is most obvious in model insects like *Drosophila melanogaster* and *Tribolium castaneum* that have only retained seven and nine Wnt genes, respectively (e.g., [39]. Other arthropods have retained representatives of most (e.g., the myriapods *Glomeris marginata* and *Strigamia maritima*, and the spider *Parasteatoda tepidariorum*) or all (the crustacean *Daphnia pulex*) of the 12 Wnt families found in arthropods [22, 39]. In spiders, however, some Wnt genes are represented by two paralogs, the result of a whole genome duplication (WGD) that took place in the lineage leading to Arachnopulmonata (e.g., spiders, whip spiders, scorpions) [46, 81].

**Fig. 1:**
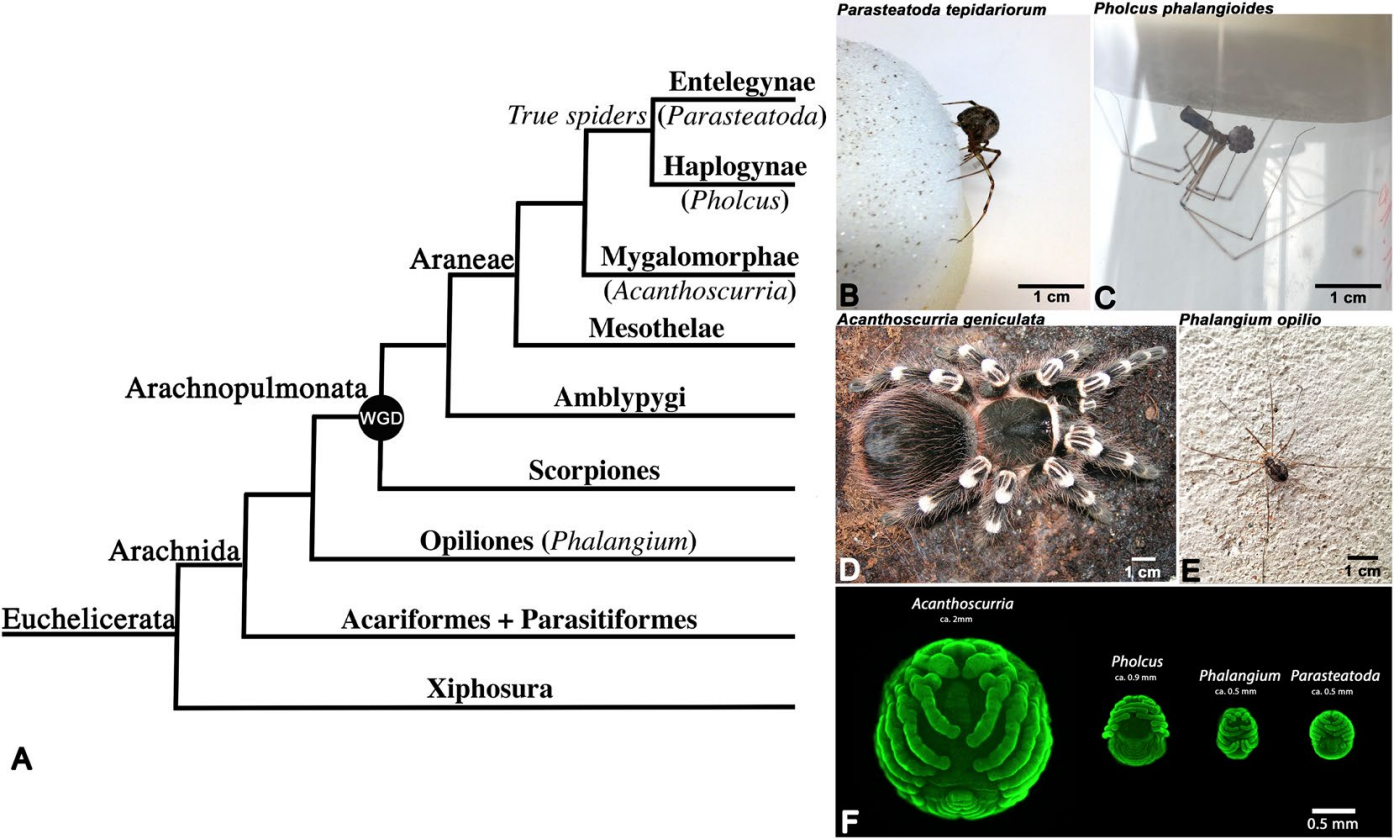
Research organisms and their embryos. **A** Chelicerate phylogeny. ‘True spiders’ (Haplogynae and Entelegynae—separated by the morphology of their female mating apparatus, represented by *Pholcus* and *Parasteatoda*, respectively). Mygalomorpha is represented by *Acanthoscurria* expanding the study towards spiders sensu lato. True spiders possess a pair of book lungs on the second opisthosomal segment (O2), a pair of tracheal tubes on O3, and spinnerets on O4 and O5. In tarantulas, book lungs develop on both, O2 and O3, and the spinnerets on O4 are rudimentary. A whole genome duplication (WGD) in the lineage leading to Arachnopulmonata is indicated. The harvestman *Phalangium* represents a chelicerate outside Arachnopulmonata, and thus a species that has not undergone a WGD. In comparison to spiders, harvestmen only have one pair of tracheal tubules on their opisthosoma (O2), and do not possess book lungs and spinnerets. **B** Adult female of the common house spider *Parasteatoda tepidariorum*. **C** Adult female of the cellar spider *Pholcus phalangioides* holding a cocoon with her chelicerae. **D** Adult female of the tarantula *Acanthoscurria geniculata*. **E** Adult female of the harvestman *Phalangium opilio* on a house wall. **E** Size comparison of the embryos of the investigated chelicerate species. The embryos are stained with the nuclear dye SYBR-green

Research on chelicerates in general and spiders in particular has greatly expanded in the last two decades providing key insights into the genomics, development, evolution, and ecology of arthropods more broadly (e.g., [11, 15, 20, 27, 57, 64, 76, 85]. However, despite the increasing interest in both Wnt-signaling and chelicerate research, we still lack truly comprehensive data about the expression profiles of Wnt genes in any chelicerate species. This includes the current main model species *Parasteatoda* in which Wnt genes have been studied rather intensively. However, also these studies do neither cover all Wnt genes nor all aspects of embryonic expression [39, 58]. In general, data on Wnt gene expression from other spider and chelicerate species are scarce. Therefore, we further explored the expression of all *Parasteatoda* Wnt genes, including those that were not investigated in previous studies. In order to establish a basis for comparative studies, we also characterized the embryonic expression profiles of all known Wnt genes in two other spiders, the cellar spider *Pholcus phalangioides* and the tarantula *Acanthoscurria geniculata* representing the haplogyne clade of araneomorphs and the mygalomorph infraorder, respectively (Fig. 1). With respect to gene duplication, the analysis revealed partially different complements of Wnt genes in these different spider lineages. Furthermore, we discovered conserved as well as divergent expression patterns of spider Wnt genes with respect to those of the harvestman *Phalangium opilio,* which did not have an ancestral WGD (Fig. 1). Our data reveal some patterns of sub- and neo-functionalization of Wnt genes after duplication and retention in spiders. More importantly, however, our data strongly suggest that Wnt gene patterning is subject to a high degree of redundancy, combinatorial function and function-shuffling (i.e., the adoption of a function of a given Wnt gene by another Wnt gene, e.g., [56, 84]. In summary, this chelicerate Wnt gene atlas highlights the complexity and evolutionary flexibility of Wnt gene expression and function. This in mind, we suggest that gene expression analyses and functional studies targeting a single (or more) Wnt gene(s) have to be interpreted with care, especially with respect to questions concerning the evolution of animals and their development.

## Methods

### Animal husbandry, embryo collection and in situ hybridization

*Parasteatoda* embryos were collected from the colony established in Uppsala, Sweden, and were treated as described in Prpic et al. [73] (Fig. 1A, B, F). Embryos of *Pholcus* were collected from wild-caught specimens in Munich and Lower Saxony, Germany, and were treated as described in Turetzek and Prpic [89] (Fig. 1A, C, F). *Acanthoscurria* embryos were collected from the established colony in Cologne, Germany, and were treated as described in Pechmann and Prpic [67] (Fig. 1A, D, F). Embryos of *Phalangium* were collected from wild-caught specimens in Uppsala, Sweden (Fig. 1A, E, F). Several males and females were kept together in large (40 L) plastic boxes. Clutches of eggs were deposited by the females into petri dishes with moistened peat moss. The embryonic chorion was dissolved in commercial bleach (Klorix) for 3–5 min followed by rinsing of the embryos in tap water. Embryos were then fixated in a 50% volume of 4% formaldehyde in phosphate buffered saline (PBS) and 50% volume heptane for 12–16 h at room temperature on a gently rocking platform. After fixation, embryos were transferred to 100% methanol and stored at − 20 ºC. Prior to in situ hybridization experiments, the vitelline membrane was removed with fine forceps. All in situ hybridizations were performed using a standardized protocol published in Janssen et al. [42]. We apply the staging system of *Parasteatoda* [60], as accurately as possible, to all here investigated species to simplify comparison of gene expression data. For further information on the different developmental stages, we refer to the original descriptions by Turetzek and Prpic [89] (*Pholcus*), Pechmann [68] (*Acanthoscurria*) and Juberthie [44] (*Phalangium*). In this study, we investigated all stages from the formation of the early germ band to dorsal closure, for *Parasteatoda* and *Pholcus*, we also investigated the earlier germ disc stage (stages 4 and 5). In the other species, this disc is unfortunately too fragile to survive the fixation and in situ hybridization procedures.

### Identification of Wnt genes

Reciprocal BLAST searches (tBLASTn) were performed against the embryonic transcriptomes of *Pholcus* [41], *Phalangium* [83] and *Acanthoscurria* [68], as well as the genome of *Parasteatoda* [81], using published arthropod and onychophoran Wnt protein sequences as baits. RNA isolation, library preparation and sequencing with Illumina HiSeq2000 for *Pholcus* was previously described [41]. The reads of the *Pholcus* transcriptome were de novo assembled after quality trimming and filtering with Trimmomatic [3] using Trinity (version r20140717, –seqType fq –JM 240 G – run_as_paired –CPU 6 [19].

Retrieved protein sequences were aligned by applying T-Coffee with default parameters in MacVector v12.6.0 (Additional file 10). Phylogenetic analysis was performed as described in Panara et al. [66], using MrBayes [31]. Sequence identifiers of all identified sequences are listed in Additional file 11.

### Gene cloning

Total RNA from *Parasteatoda* and *Phalangium* was isolated from a mix of embryonic stages using TRIzol (Invitrogen). For *Phalangium*, we isolated mRNA from total RNA using the Dynabeads mRNA Purification Kit (Invitrogen) followed by reverse transcription into cDNA (SuperScriptII first-strand synthesis system for RT-PCR, Invitrogen). For *Pholcus* and *Acanthoscurria*, RNA isolation and cDNA synthesis were carried out as previously described [90] (*Pholcus*), [68] (*Acanthoscurria*)). Genes were amplified using RT-PCR with gene-specific primers (in most cases a second/nested PCR was performed using a second set of primers and the first PCR as template). For *Pholcus*, some Wnt genes were isolated using gene-specific primers in combination with degenerate primers, (Additional file 12). Gene fragments obtained were cloned into pCR-II or pCR2.1 (TA Cloning Kit Dual Promoter, Invitrogen) or Pjet1.2 (CloneJET PCR Cloning Kit), and sequenced using the commercial sequencing services offered by Macrogen or Eurofins Genomics.

### Data documentation

Staining of embryos was either documented from whole mounts, in the form of flat-mounted parts of the embryos, or in the form of dissected appendages. For the dissection of appendages, we used fine tungsten needles recycled from burned-out old-fashioned light bulbs that were sharpened in the flame of a Bunsen burner.

Bright field microscopy and visualization of the nuclear dye SYBR-green were performed under a MZ-FLIII Leica dissection microscope using a Leica DC490 digital camera equipped with an external UV-light source. Whenever necessary and appropriate, linear adjustments were performed on color, contrast and brightness with the image-processing software Adobe Photoshop CC 2018.

## Results

### Wnt genes in spiders and a harvestman

We reanalyzed the Wnt gene repertoire of *Parasteatoda* and surveyed the repertoires of these genes in additional spiders, *Pholcus* and *Acanthoscurria,* as well as the harvestman *Phalangium* screening embryonic transcriptomes of all species and the genome of *Parasteatoda*. Our phylogenetic analysis was similar to those by Harper et al. [21].

**Fig. 2:**
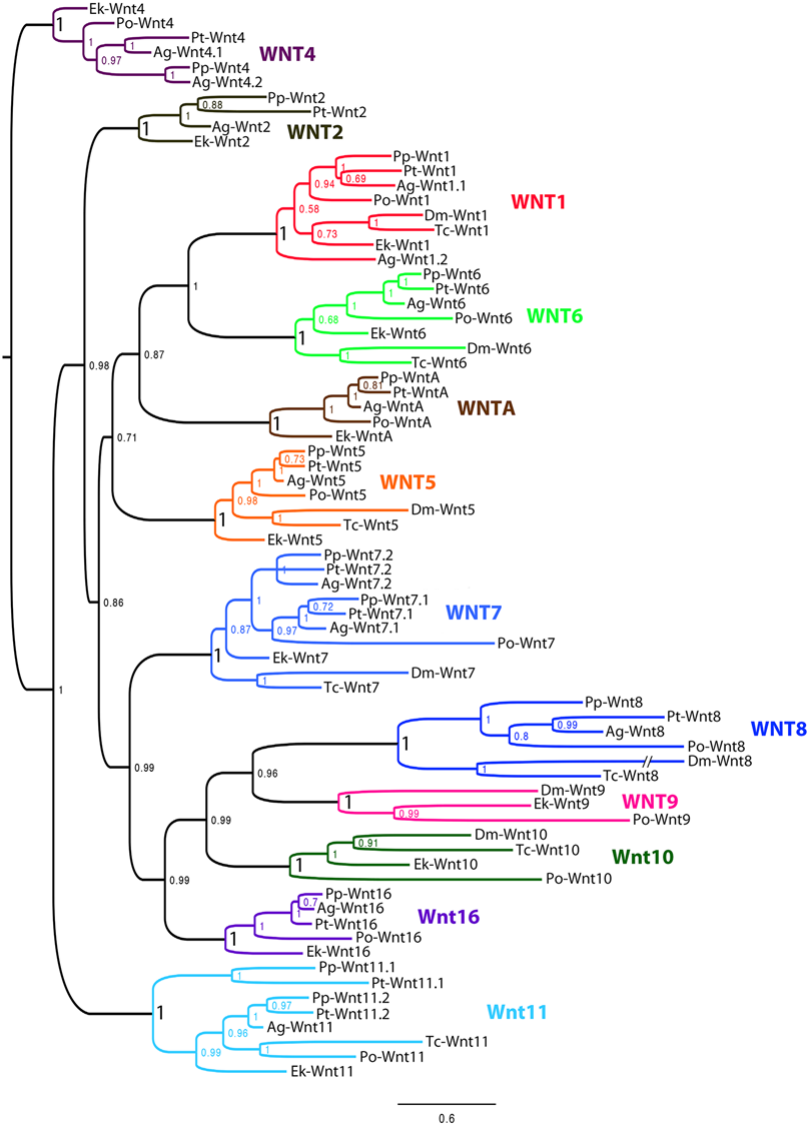
Phylogenetic analysis of Wnt genes in spiders and a harvestman. Species abbreviations: Ag, *Acanthoscurria geniculata*; Dm, *Drosophila melanogaster* (Hexapoda: Diptera); Ek, *Euperipatoides kanangrensis* (Onychophora); Po, *Phalangium opilio*; Pp, *Pholcus phalangioides*; Pt, *Parasteatoda tepidariorum*; Tc, *Tribolium castaneum* (Hexapoda: Coleoptera). Node support is given as posterior probabilities. Note that all classes of Wnt genes cluster with absolute support

**Fig. 3:**
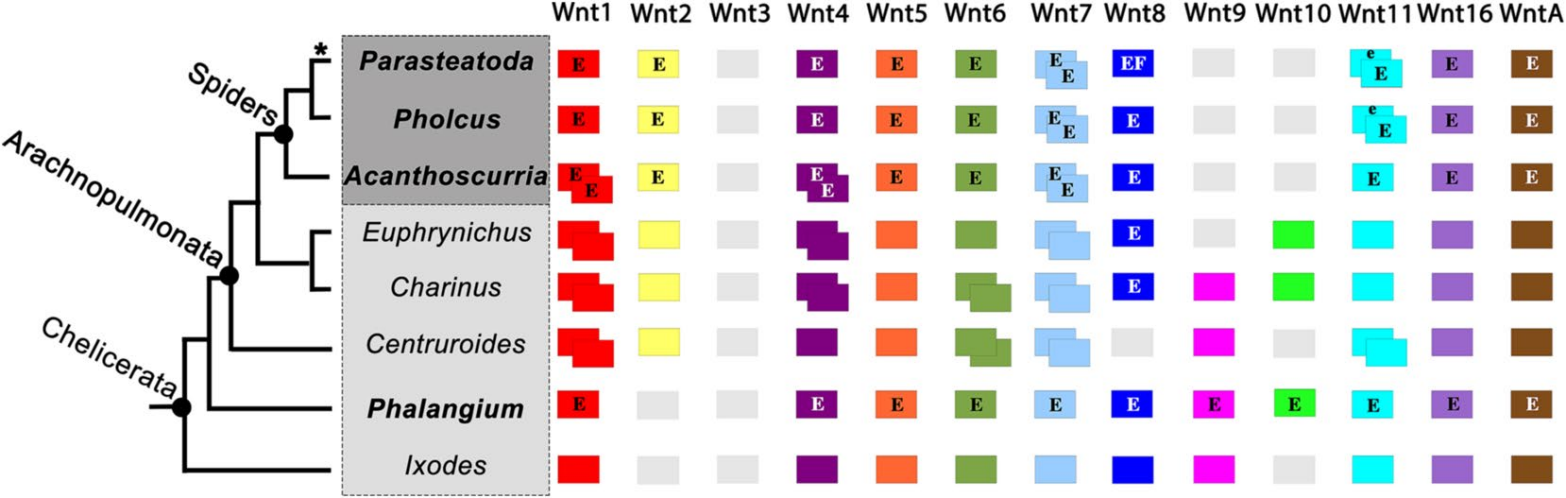
The Wnt gene complements of spiders and a harvestman. Note the duplicated Wnt genes in arachnopulmonate chelicerates compared to non-arachnopulmonate chelicerates. The asterisk (*) indicates that the complement of other entelegyne spiders is identical with that of *Parasteatoda* with the exception that a second *Wnt4* paralog has been retained. Dark grey box marks spiders, light grey box marks non-spider chelicerates. An expanded overview over Wnt gene complements in Panarthropoda is provided in Additional file 1: Fig. S1. Full species names and sources of gene content: *Acanthoscurria geniculata* (embryonic transcriptome); *Centruroides sculpturatus* (genome); *Charinus acosta* (embryonic transcriptome); *Euphrynichus bacillifer* (embryonic transcriptome); *Ixodes scalpularis* (genome); *Parasteatoda tepidariorum* (genome and embryonic transcriptome); *Phalangium opilio* (embryonic transcriptome); *Pholcus phalangioides* (embryonic transcriptome). Two overlaying boxes indicate the presence of two orthologs. The figure is based on previously published data and the color code follows these studies (e.g., [21, 28, 39]). Abbreviations: e, expression has been studied, but no specific signal has been reported; E, expression has been studied; F, functional studies are available

The common ancestor of chelicerates likely possessed a “complete” set of the 12 Wnt genes typical for protostomes, despite common lineage-specific losses within this subphylum (Figs. 2, 3; Additional file 1: Fig. S1) [21, 39]. Spiders appear to have lost their *Wnt9* and *Wnt10* orthologs, while these genes are retained in other chelicerates such as the harvestman *Phalangium* [21]. Two paralogs of *Wnt1, Wnt7*, and *Wnt11* have been retained (after the WGD in Arachnopulmonata) but the second paralog of *Wnt1* has been lost in most true spiders [21]. The lack of a second *Wnt4* paralog in *Pholcus* and the presence of two paralogs of *Wnt4* in *Acanthoscurria*, as well as some lineages of entelegyne spiders [21] suggest that loss of a second *Wnt4* gene occurred independently in at least two lineages of spiders (towards *Parasteatoda*, and towards *Pholcus*) (Figs. 2, 3). The apparent loss of a second *Wnt4* gene in *Pholcus* may be representative for Haplogynae as a whole as we could not identify a second copy in the published genome of another basally branching haplogyne spider, the recluse *Loxosceles reclusa* (data not shown). Please note that the lack/loss of a gene is difficult to prove, even in the era of full genome sequencing. Most genomes, although “sequenced” are not complete, or have not been assembled completely. The situation in spiders is even more complicated because of the many duplicated genes and often enlarged intronic regions. Many of the published spider genomes are thus far from having the complete set of genes. The usage of transcriptomic data (as used in our study), using a combination of sequencing methods as well as several rounds of reannotations helps to improve these issues. That is why the *Parasteatoda* genome is still one of the best annotated genomes present.

### Wnt1

**Fig. 4:**
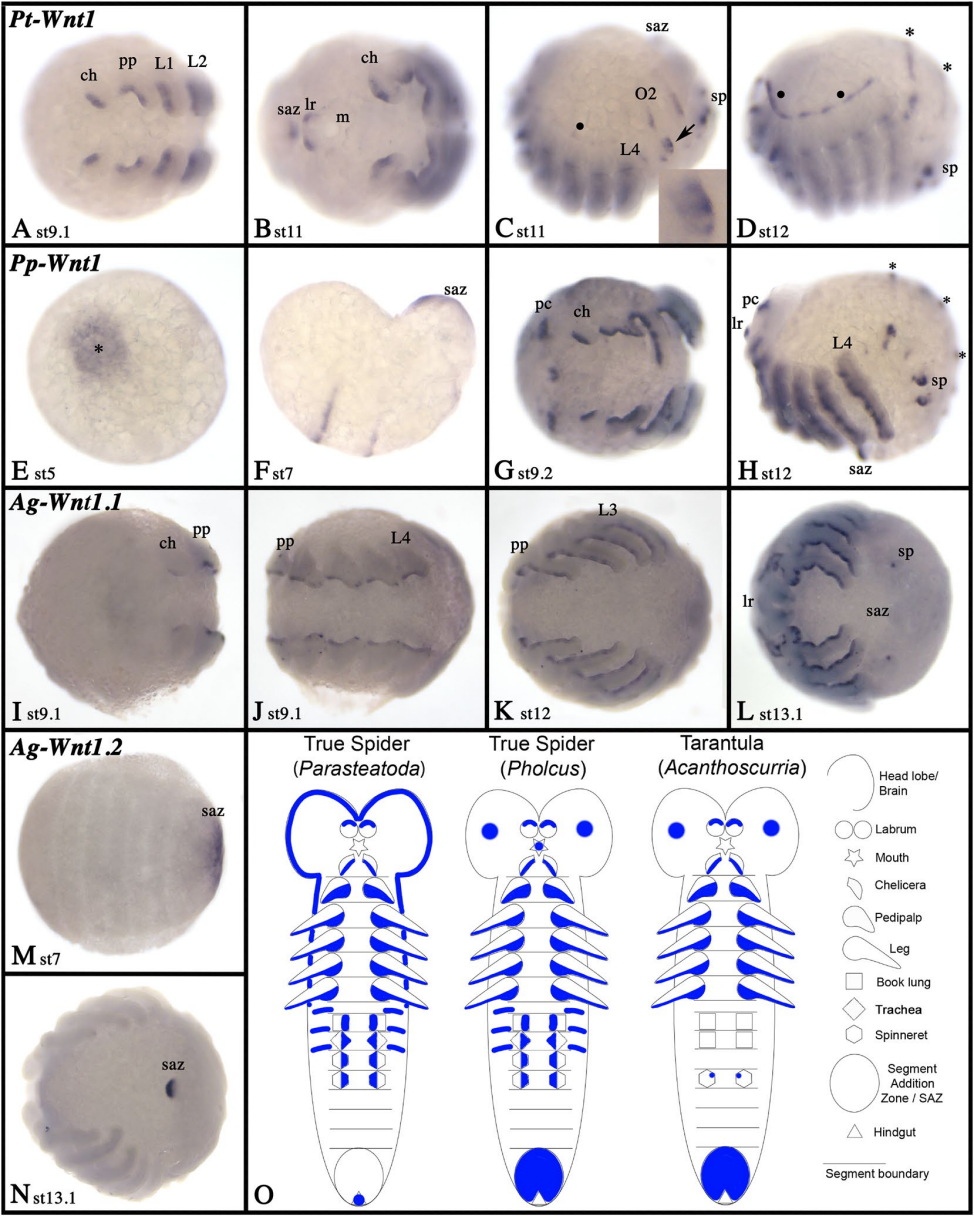
Expression of *Wnt1* genes in spiders. Expression of *Wnt1* in *Parasteatoda* (**A**–**D**), *Pholcus* (**E**–**H**), and *Acanthoscurria* ((**I**–**L** (*Wnt1.1*), **M**, **N** (*Wnt1.2*)). In all panels, except panel **O**, anterior is to the left. Ventral views, except panels **C**, **D**, **F** and **H** (lateral views). Developmental stages are indicated. Filled circles (•) in panels **C** and **D** mark expression along the dorsal rim of the prosoma. Asterisks in panel **E** mark the center of the germ disc (the later posterior region of the germ band). Asterisks in panels **D** and **H** mark dorsal stripes of expression. The arrow in panel **C** points to the book lung that expresses *Wnt1* in the form of three separate domains (cf. inlay in panel **C**). Panels indicated with an apostrophe (´) represent SYBR-green stained embryos corresponding to the embryo shown in the panel without apostrophe. Expression patterns are summarized in panel **O**, anterior is up. Abbreviations: ch, chelicera; L, leg; lr, labrum; m, mouth; O, opisthosomal segment; pc, pre-cheliceral region; pp, pedipalp; saz, segment-addition zone; sp, spinneret

**Fig. 5:**
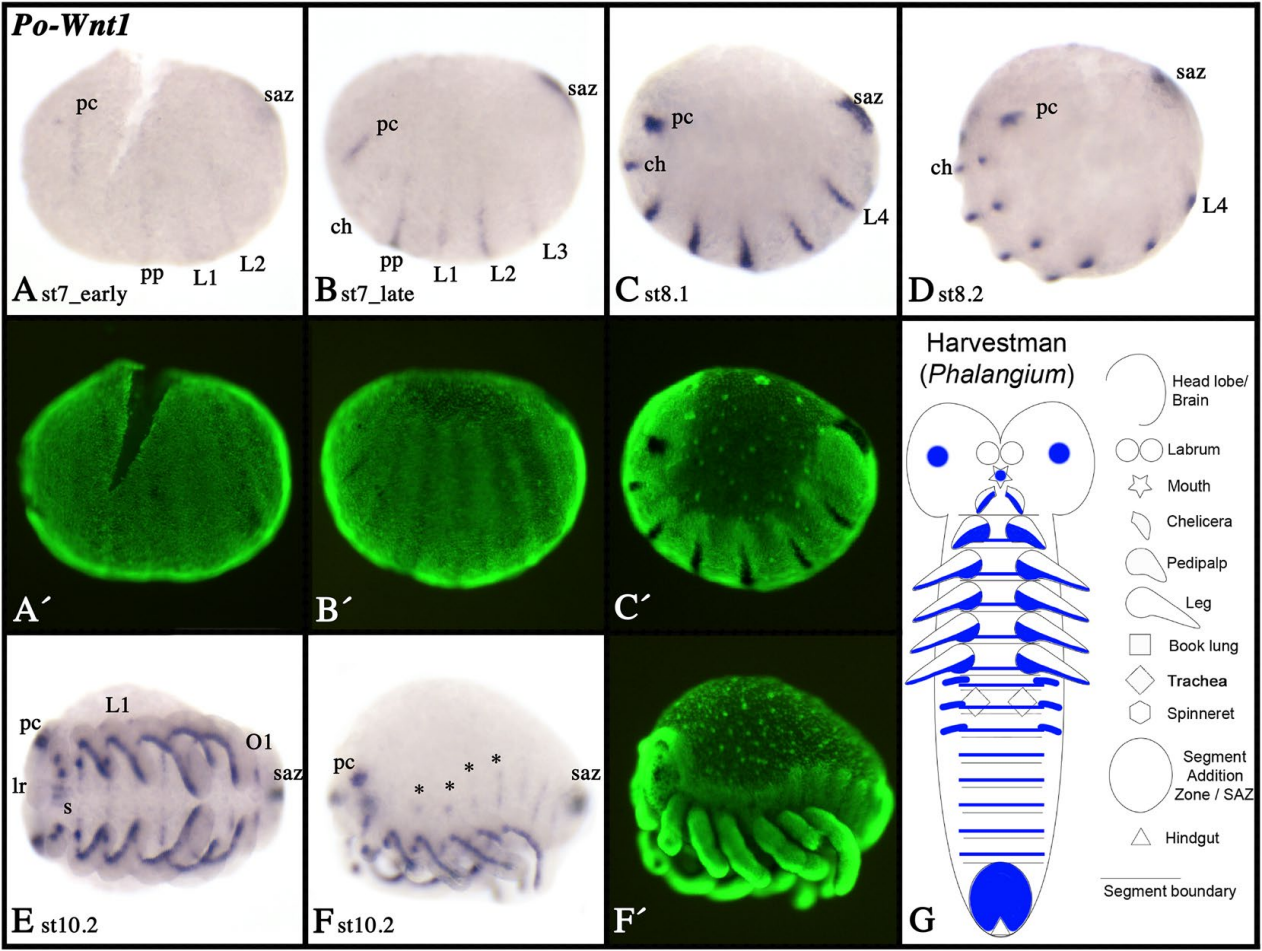
Expression of harvestman *Wnt1*. In all panels, anterior is to the left. Panels **A**–**D** and **F** show lateral views. Panel **E** shows ventral view. Developmental stages are indicated. Asterisks (*) in panel **F** mark expression in the dorsal region of the embryo. Note the segment-polarity gene like expression in the form of transverse segmental stripes. Panels **B´** represent SYBR-green stained embryo as shown in panel **B**. Expression patterns are summarized in panel **G**, anterior is up. Abbreviations as in Fig. 4, and s, stomodaeum

In all investigated species, at least one paralog of *Wnt1* is expressed in a subset of cells in the pre-cheliceral region, along the ventral side of the appendages (including the opisthosomal limb buds that correspond to the breathing organs and the spinnerets), dorsally in the labrum (except for the harvestman), and in the posterior of the developing embryo (Figs. 4, 5; Additional files 2, 3, 5, 6, 7: Figures S2, S3, S5–7). The posterior expression is either corresponding to the hindgut primordium that is located posterior to the segment-addition zone (marked with SAZ), or the posterior part of the SAZ. While this expression appears early during development in other spiders suggesting a role as posterior patterning gene, in *Parasteatoda* this expression is restricted to later developmental stages indicating that it may indeed correspond to the hindgut rather than be involved in segment addition (Fig. 4B, C). Interestingly, there are two paralogs of *Wnt1* in *Acanthoscurria*. The second paralog, *Wnt1.2*, is exclusively expressed in the SAZ (Fig. 4M, N), while the other paralog, *Wnt1*, is expressed similar to the single *Wnt1* gene in the other species, but is lacking expression in the SAZ (Fig. 4I–L). This represents an impressive example of sub-functionalization after WGD. With the exception of *Acanthoscurria*, for all species studied dorsal stripes of expression appear in the opisthosoma late during embryogenesis (Figs. 4, 5). Only in *Parasteatoda*, there is a line of expression dorsal in the head and the limb-bearing segments (Fig. 4C, D).

In true spiders, *Wnt1* is expressed in the form of segment polarity gene (SPG)-like transverse stripes, but such stripes are restricted to some of the head segments [39] (Fig. 4F). In *Acanthoscurria*, there are no SPG-like stripes of expression (Fig. 4I–N). In *Phalangium*, however, SPG-like stripes are present early during development, and in all developing segments (including posteriorly added segments) (Fig. 5A–F). Note that expression of *Wnt1* in the developing books lungs of *Parasteatoda* is in the form of three separate domains as previously described for another entelegyne spider, *Cupiennius salei* [9] (Fig. 4C(inlay)). Expression patterns of spider and harvestman *Wnt1* genes are summarized in schematic Figs. 4O and 5G, respectively.

### Wnt2

**Fig. 6:**
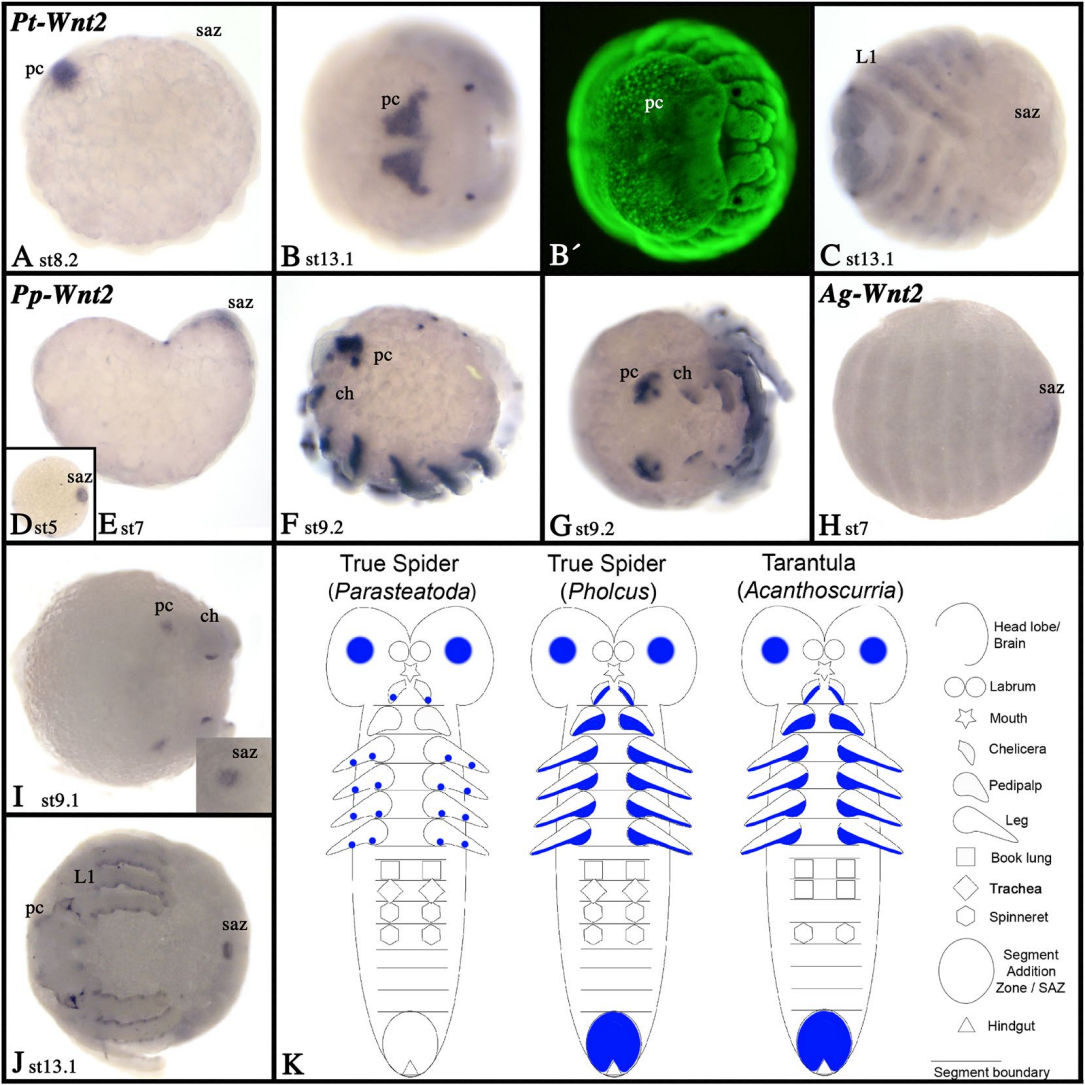
Expression of *Wnt2.* Expression of *Wnt2* in *Parasteatoda* (**A**–**C**), *Pholcus* (**D**–**G**), and *Acanthoscurria* (**H**–**J**). In all panels, anterior is to the left. Panels **A**, **E**, and **F** show lateral views, panels **B**, **G**, and **I** show anterior views. Panels **C**, **D**, **H** and **J** show lateral views. Developmental stages are indicated. Panel **B´** shows a SYBR-green staining of the embryo shown in **B**. Expression patterns are summarized in panel **G**, anterior is up. Abbreviations as in Fig. 4

We identified a single *Wnt2* ortholog in all spider species, but not in the harvestman. In all spiders, *Wnt2* is expressed in a subset of cells in the pre-cheliceral region (Fig. 6; Additional file 3, 6, 7: Fig. S3B, S6B, S7B). Notably, this domain appears already during early germ band stages in *Parasteatoda* and covers a larger area of the brain in later stages compared to *Pholcus* and *Acanthoscurria,* the latter displaying the smallest brain expression domain (Fig. 6). In *Pholcus* and *Acanthoscurria*, *Wnt2* is expressed in the SAZ throughout development, but in *Parasteatoda*, there is no such posterior expression (Fig. 6). Similarly, in *Pholcus* and *Acanthoscurria Wnt2* is expressed along the ventral side of the prosomal appendages (except for the labrum), but in *Parasteatoda* expression is restricted to some dot-like domains along the ventral side of the appendages (Fig. 6; Additional files 3, 6, 7: Figs. S3, S6, S7). Expression of spider *Wnt2* genes is summarized in the schematic Fig. 6K.

### Wnt4

In most spiders, there are two paralogs of *Wnt4* [21], in *Pholcus* and *Parasteatoda*, however, only one *Wnt4* is present (*Parasteatoda*) or has been identified in an embryonic transcriptome (*Pholcus*) (Fig. 3). Only in *Acanthoscurria*, we were able to identify two paralogs of *Wnt4*.

**Fig. 7:**
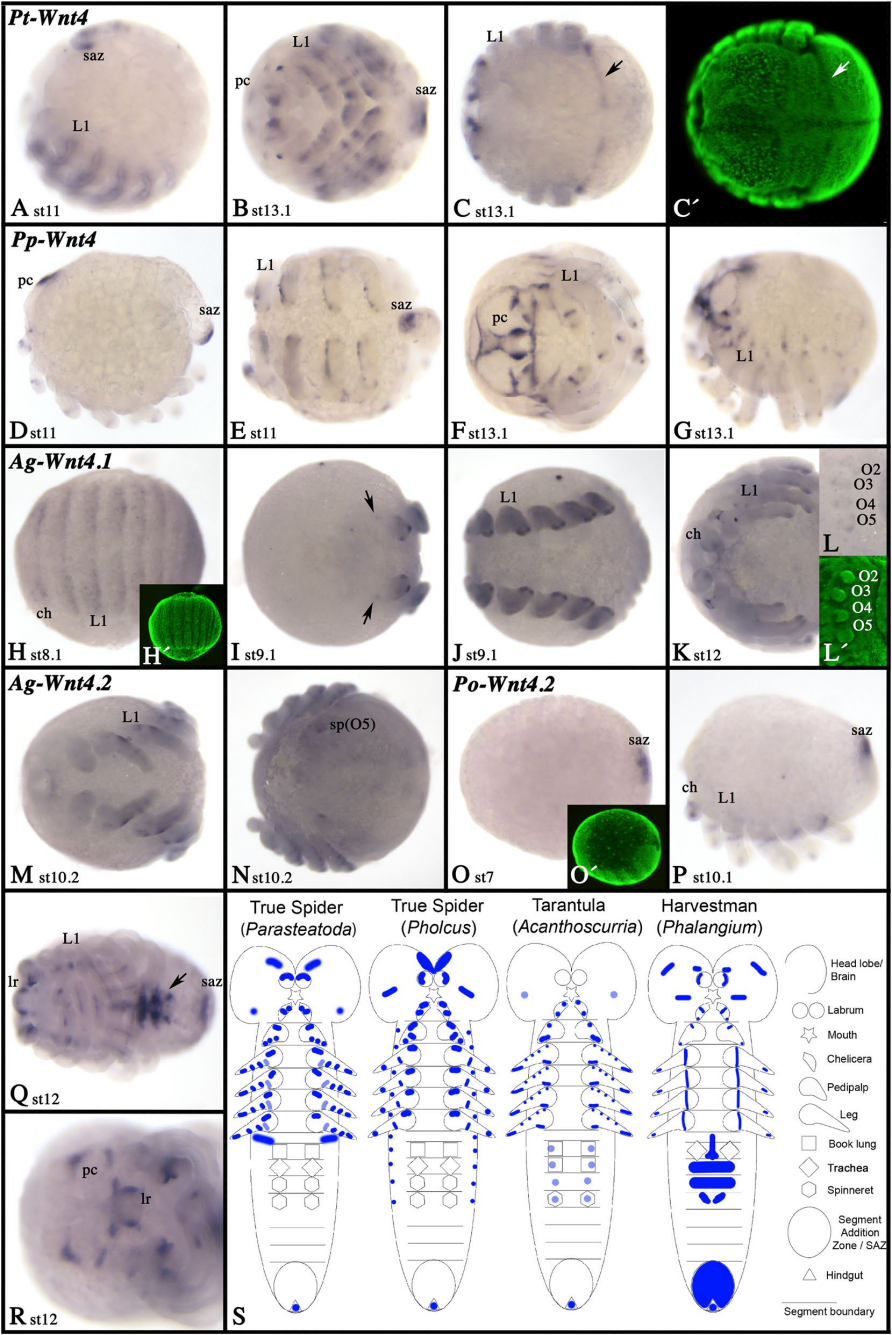
Expression of *Wnt4* genes. Expression of *Wnt4.1* in *Parasteatoda* (**A**–**C**), *Pholcus* (**D**–**G**), *Acanthoscurria* (**H**–**L** (*Wnt4.1*), M, N (*Wnt4.2*)) and *Phalangium* (**O**–**R**). In all panels, anterior is to the left. Panels **A**, **D**, **G**, **O** and **P** present lateral views. Panels **B**, **E**, **H**, **J**–**M**, and **Q** show ventral views. Panel **C** presents a dorsal view. Panels **F** and **R** show anterior views, and panels **N** shows a posterior view. Panel **L** shows magnification of the opisthosomal limb buds (same embryo as panel **K**). Panels **C´**, **H´**, **L´** and **O´** show SYBR-green staining of the embryo shown in corresponding bright field panels. Developmental stages are indicated. The arrow in panel **C** points to a dorsal stripe of expression that separates pro- and opisthosoma. The arrows in panel I point to weak and small domains in the pre-cheliceral region. The arrow in panel **Q** points to expression in the ventral region of the opisthosoma. Expression patterns are summarized in panel **S**, anterior is up. Abbreviations as in Fig. 4

*Wnt4* exhibits quite diverse expression among spiders and between these animals and the harvestman (Fig. 7; Additional files 3, 5, 6, 7: Fig. S3, S5–S7). The only common features are the dot-like domains in the distal ectoderm of the legs and pedipalps of spiders, and the expression in the labrum (except for *Acanthoscurria*). In the harvestman, however, expression in the pedipalps and legs is different to the spiders and restricted to a distal portion of the limb mesoderm (cf. Additional files 3, 5, 6, 7: Fig. S3, S5–S7). Although expression in the legs and pedipalps of spiders is mainly restricted to ventral tissue, one of the two tarantula *Wnt4* genes (*Wnt4.2*) is expressed in dorsal (and rather proximal) domains (cf. panels C and D of Additional file 3: Figure S3). Patterns of presence and absence in the prosomal appendages of spiders differs between the investigated species (Additional files 3, 5, 6, 7: Figs. S3, S5–S7). In all species (except *Acanthoscurria*), there is a complex pattern of expression in the pre-cheliceral region (Fig. 7). In all species, *Wnt4* is expressed at the posterior pole of the developing embryo, although the signal in *Acanthoscurria* is very weak and thus may represent background (Fig. 7). In true spiders, expression in the posterior is clear, but only appears at relatively late developmental stages, while comparative expression appears very early during germ band formation in the harvestman (Fig. 7O). Only in the tarantula, one of the two *Wnt4* paralogs (*Wnt4.1*) is expressed in SPG-like stripes early during development (Fig. 7H), and in the harvestman a unique ventral expression appears during later stages in the opisthosoma (Fig. 7Q). Another unique expression is present for *Parasteatoda Wnt4* forming a dorsal stripe separating the prosoma from the opisthosoma (Fig. 7C). The expression patterns of *Wnt4* genes are too diverse to identify possible patterns of sub- or neo-functionalization in *Acanthoscurria*. Here, expression patterns of other chelicerate species that retained two paralogs could help to clarify an ancestral feature of *Wnt4*. Expression patterns of *Wnt4* genes are summarized in the schematic Fig. 7S.

### Wnt5

**Fig. 8:**
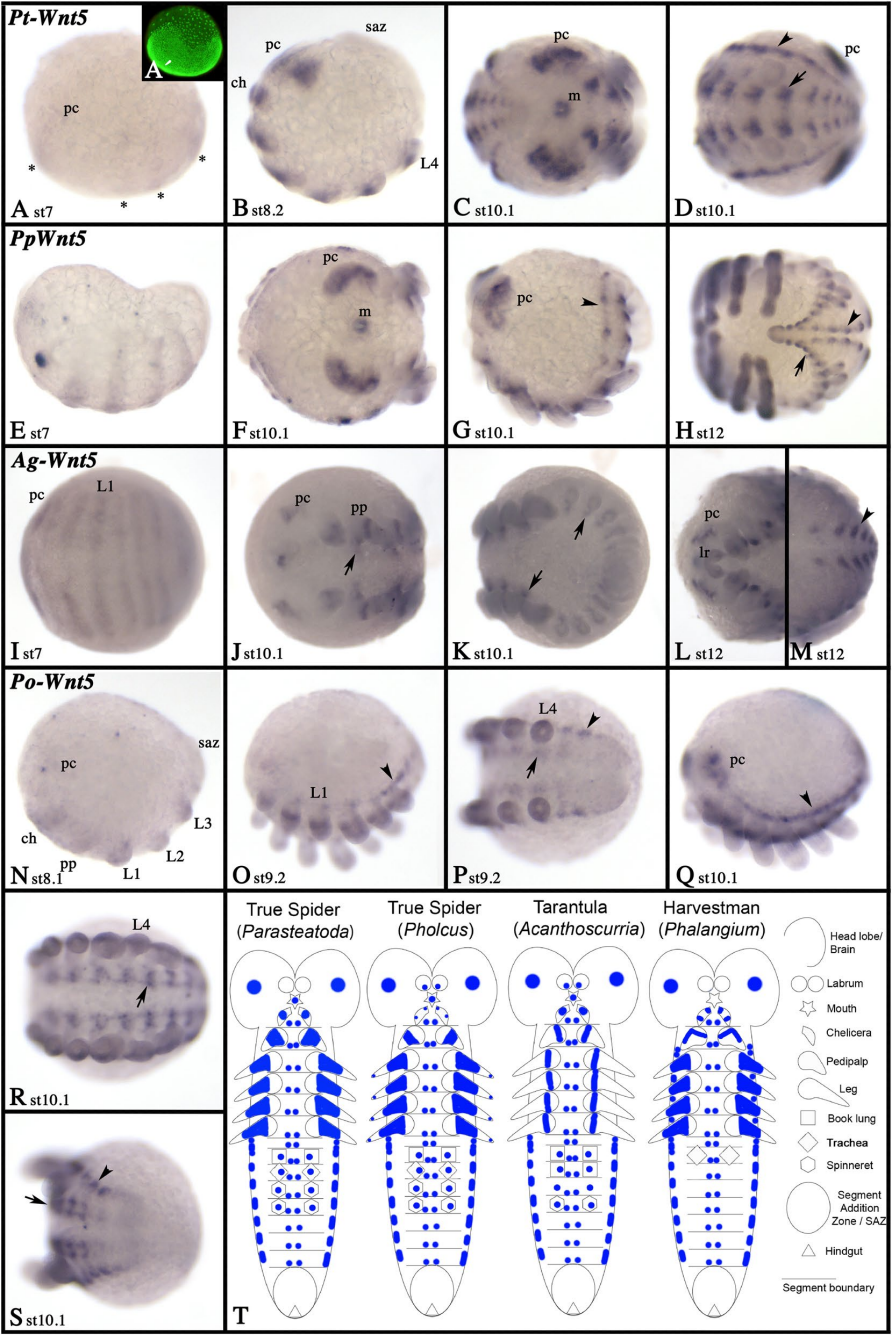
Expression of *Wnt5* genes. Expression of *Wnt5* in *Parasteatoda* (**A**–**D**), *Pholcus* (**E**–**H**), *Acanthoscurria* (**I**–**M**) and *Phalangium* (**N**–**S**). In all panels, anterior is to the left. Panels **A**, **B**, **E**, **G**, **N**, **O**, and **Q** show lateral views. Panels **C**, **D**, **H**, **I**–**L**, **P**, **R**, and **S** show ventral views. Panel **M** represents a dorsal view. Panel **A´** represent SYBR-green staining of the embryo shown in panels **A**. Developmental stages are indicated. Asterisks in panel **A** mark faint stripes of expression. In all panels, arrows and arrowheads point to expression in the ventral nervous system and the heart, respectively. Expression patterns are summarized in panel **T**, anterior is up. Abbreviations as in Fig. 4

In *Parasteatoda* and *Pholcus*, expression of *Wnt5* starts after germ band formation and shortly before the limb buds begin to grow out (Fig. 8A, E). The same pattern is seen in the early germ bands of *Acanthoscurria* and *Phalangium*, but we do not know if expression starts already earlier in these species (Fig. 8I, N). This expression most likely correlates with the limb primordia. Furthermore, in all species, *Wnt5* is expressed in a large domain of the pre-cheliceral region and the ventral nervous system (Fig. 8; Additional files 5, 7: Figs. S5C, S7D). *Wnt5* is also expressed in all appendages, including the opisthosomal limb buds, but not in the labrum (with the exception of dot-like domains late in *Acanthoscurria* and *Pholcus*) (Fig. 8; Additional files 3, 5, 7: Figs. S3, S5–S7). Interestingly, in all species, the limb expression resembles leg-gap gene like domains. In all species, *Wnt5* is also expressed in the dorsum of the opisthosomal segments; likely, this expression is correlated with the development of the heart (arrowhead in Figs. 8D, G, H, M, O–Q, S) (cf. [37]). In the three spiders, but not in the harvestman, *Wnt5* is also expressed is in the stomodeum (Fig. 8C, F, J; Additional file 7: Figure S7D). *Wnt5* expression is summarized in the schematic Fig. 8T.

### Wnt6

**Fig. 9:**
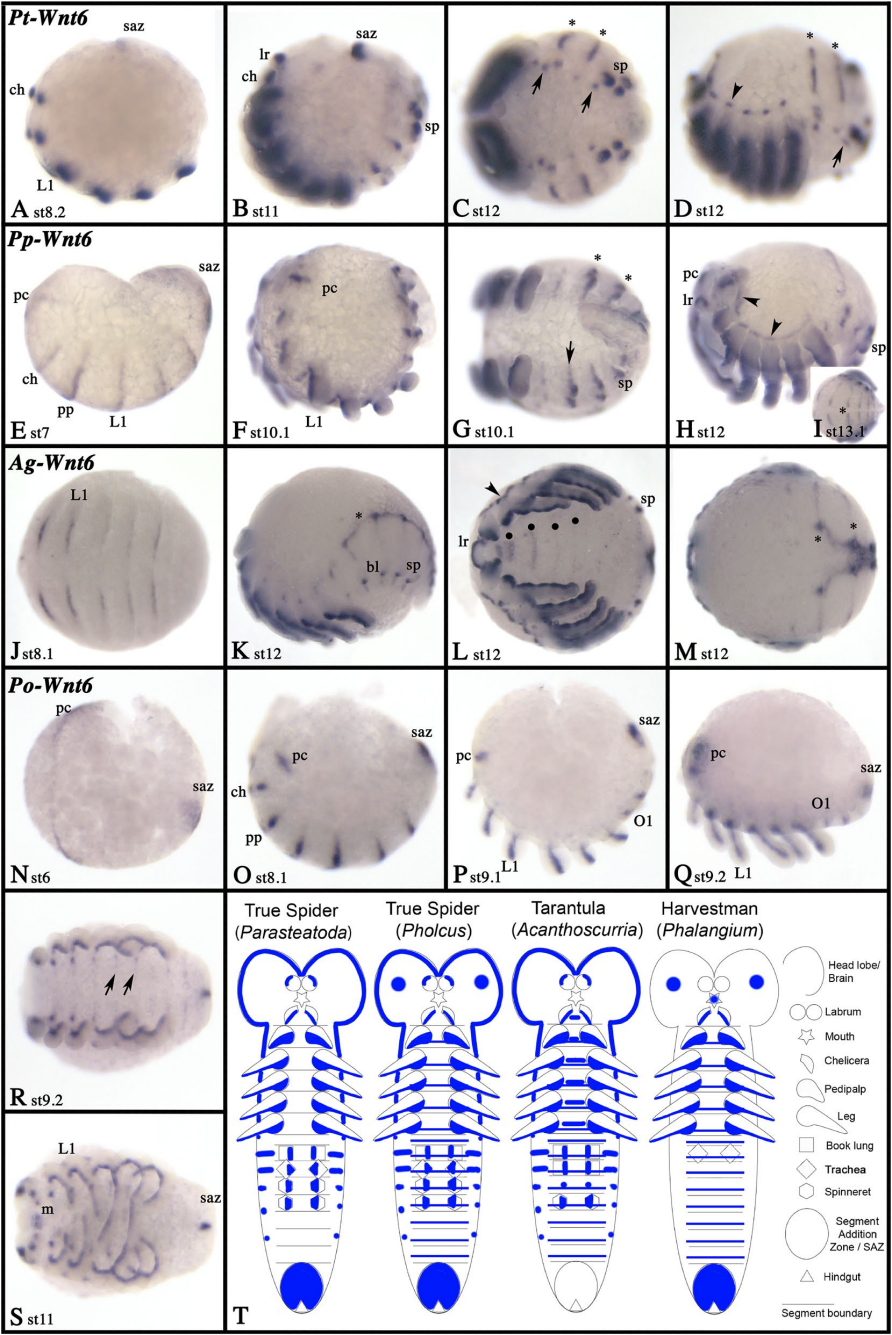
Expression of *Wnt6* genes. Expression of *Wnt6* in *Parasteatoda* (**A**–**D**), *Pholcus* (**E**–**I**), *Acanthoscurria* (**J**–**M**) and *Phalangium* (**N**–**S**). Panels **C**, **G**, **J**, **L**, **N**, **R** and **S** show ventral views. Panels **I** and **M** show dorsal views. Panels **A**, **B**, **D**, **E**, **F**, **H**, **K**, and **O**–**Q** show lateral views. Developmental stages are indicated. In all panels, asterisks (*) mark dorsal stripes of expression and expression in the forming heart, arrows and arrowheads point to expression in the ventral nervous system and the dorsal rim of the prosoma, respectively. Filled circles in panel **U** mark segmental stripes of expression in the ventral sulcus. Expression patterns are summarized in panel **T**, anterior is up. Abbreviations as in Fig. 4; and bl, book lungs

In all species, *Wnt6* is expressed along the ventral side of all appendages, including the opisthosomal limb buds (Fig. 9; Additional files 3, 5, 6, 7: Figs. S3, S5–7). In the labrum, *Wnt6* is expressed dorsally but note that *Phalangium Wnt6* is not expressed in the labrum at all (Fig. 9H, L; Additional files 5, 7: Figs. S5D, S7E). In *Parasteatoda,* expression starts when the limb buds begin to grow out (Fig. 9A). In *Acanthoscurria*, the earliest *Wnt6* expression commences just before the formation of the limb buds in a SPG-like fashion (Fig. 9J). In *Pholcus* and *Phalangium*, expression starts earlier and in SPG-like transverse stripes before the onset of limb bud development (Fig. 9E, N). The anterior-most stripe is correlated with later expression in the pre-cheliceral region. This expression was not observed in *Parasteatoda* or *Acanthoscurria* (Fig. 9). The early stripes later become restricted to expression in the developing appendages and thin stripes of expression ventral to the base of the appendages, most prominently seen in *Phalangium* where the germ band halves do not split unlike in spiders (Fig. 9R). In *Phalangium* and *Parasteatoda*, *Wnt6* is expressed in the SAZ, but while this expression is already present in early stages of the harvestman, expression in this spider appears later during germ band extension (Figs. 9A, N). The other two spiders do not express *Wnt6* posteriorly (Fig. 9), except for an early transient posterior domain in *Pholcus* (Fig. 9E). In all spiders, *Wnt6* is also expressed dorsal to the base of the appendages, which is especially prominent in the opisthosoma (Fig. 9). This expression is likely correlated with the development of the heart and in *Acanthoscurria*, the developing heart tube itself expresses *Wnt6* (Fig. 9K, M). Additional expression of *Wnt6* was observed in the stomodaeum of the harvestman (Fig. 9S), and in the form of transverse segmental stripes in the ventral sulcus (the region between the split germ band halves) of the tarantula (Fig. 9L). Similar stripes of expression in the ventral sulcus have been reported for *netrin* expression in spiders including *Parasteatoda*, suggesting that *Wnt6* may be involved in axonal guidance [48]. Expression of *Wnt6* is summarized in the schematic Fig. 9T.

### Wnt7

**Fig. 10:**
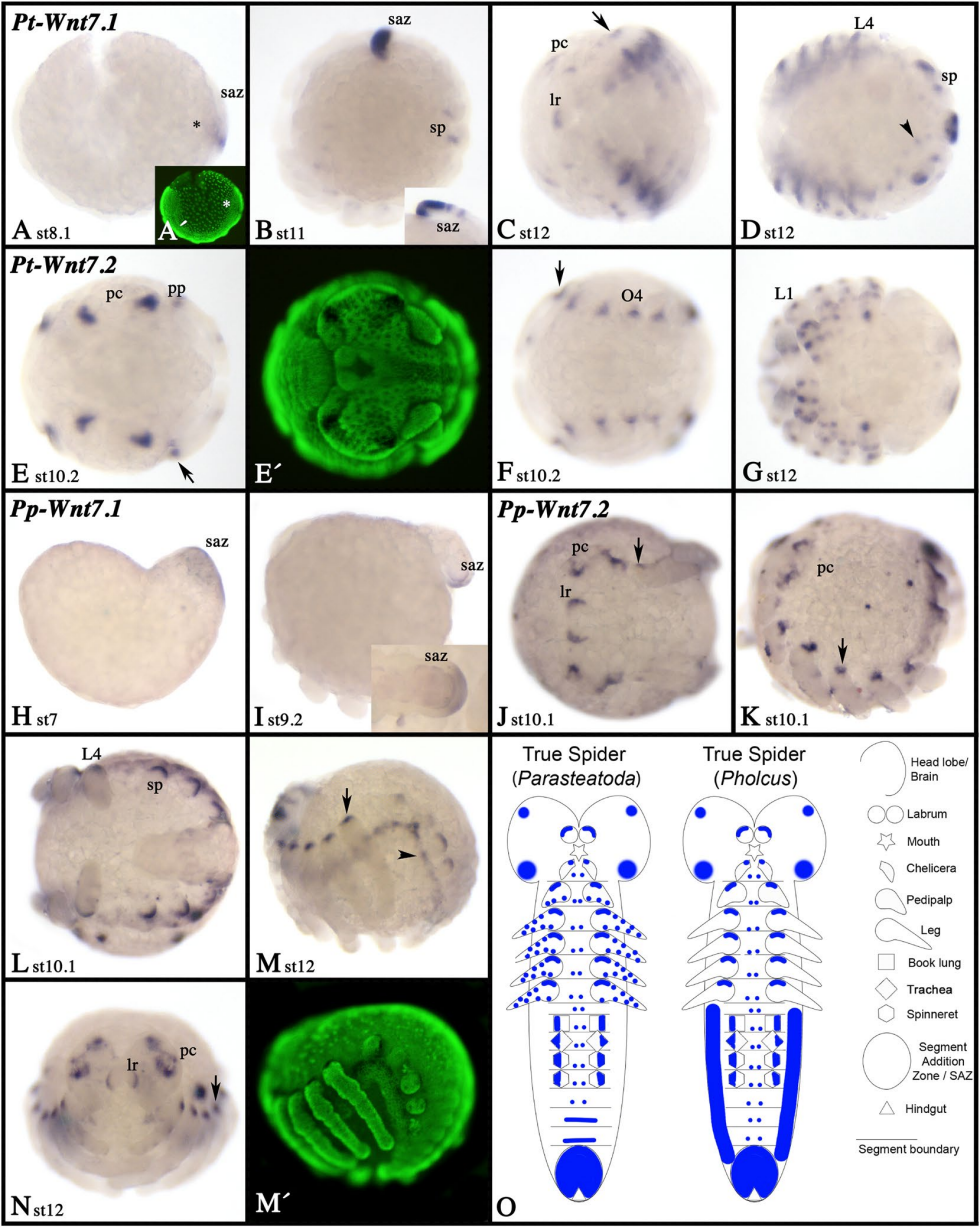
Expression of *Wnt7* genes in true spiders. Expression of *Wnt7* in *Parasteatoda* (**A**–**D** (*Wnt7.1*), **E**–**G** (*Wnt7.2*)), and  *Pholcus* (**H**, **I** (Wnt7.1), **J**–**N** (*Wnt7.2*)). In all panels (except panel **N**), anterior is to the left. Panels **A**, **B**, **H**, **I**, **K** and **M** show lateral views. Panels **C**, **D**, **F**, **G**, **J** and **L** ventral views. Panels **E** and **N** show anterior views; in panel **N** anterior is up. The inlay in panel **B** shows the SAZ of a slightly older embryo. Panels indicated with an apostrophe represent SYBR-green staining of the embryos in corresponding panels. Developmental stages are indicated. In all panels, arrows mark expression dorsal to the base of the limbs. The arrowhead in panel **M** points to expression in the ventral nervous system. Expression patterns are summarized in panel **O**, anterior is up. Abbreviations as in Fig. 4

**Fig. 11:**
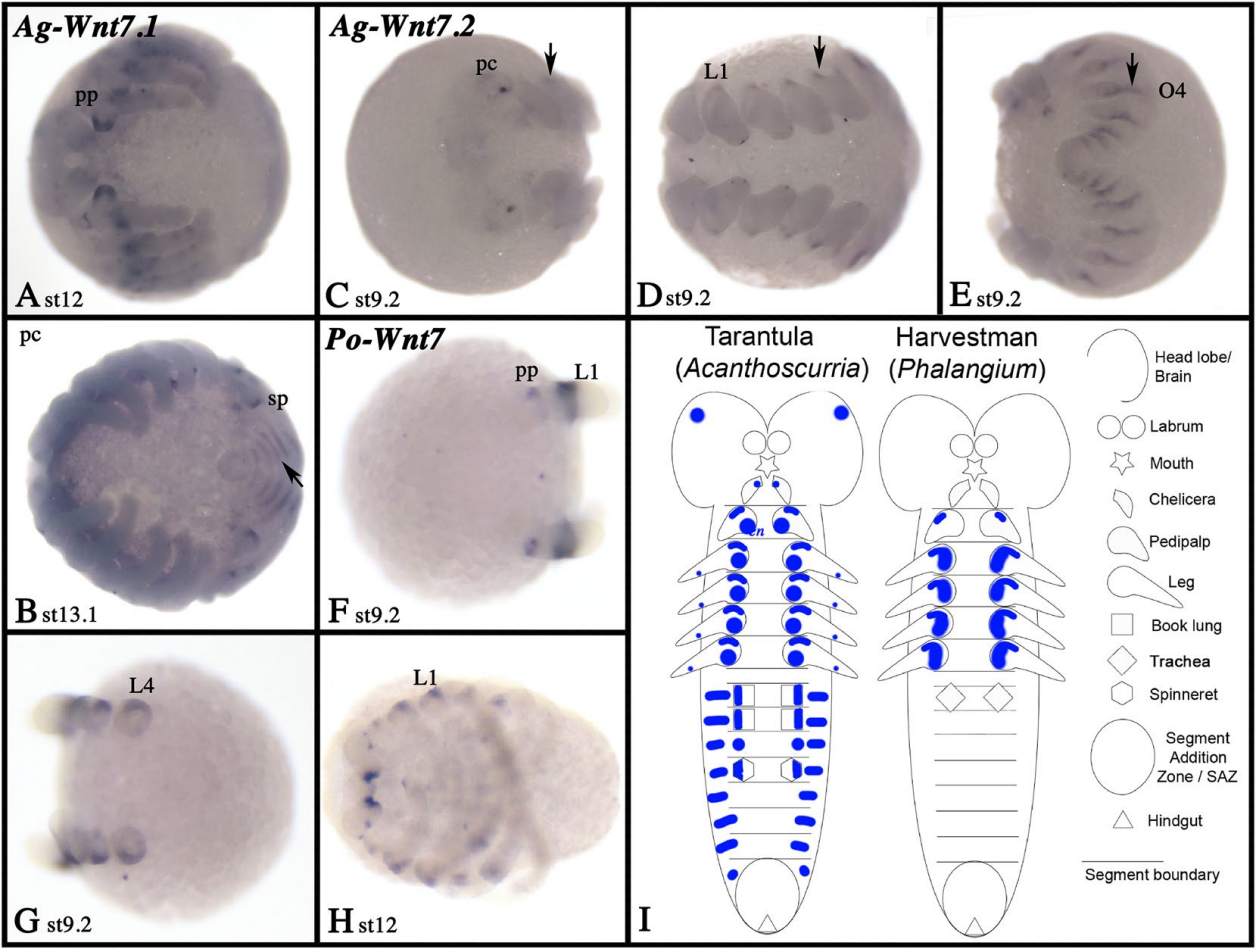
Expression of *Wnt7* genes in the tarantula and the harvestman. Expression of *Acanthoscurria Wnt7.1* (**A**, **B**) and *Wnt7.2* (**C**–**E**), and *Phalangium Wnt7* (**F**–**H**). In all panels, anterior is to the left. Panels **A**, **C**, and **F** present anterior views, the other panels show ventral views. Developmental stages are indicated. Arrows point to expression dorsal to the base of the limbs. Expression patterns are summarized in panel **I**, anterior is up. Abbreviations as in Fig. 4

All spiders investigated here possess two *Wnt7* paralogs (Fig. 3). In true spiders, one *Wnt7* gene (*Wnt7.1*) is expressed in the posterior SAZ region (Fig. 10A, B, D, H, I). While this is the only expression of *Wnt7.1* observed in *Pholcus*, *Parasteatoda Wnt7.1* is also expressed in the developing limb buds including the opisthosomal buds, and in part of the brain and the ventral nervous system (Fig. 10B–D). In the limbs, this expression is predominantly present along the ventral side, but a dot of expression is also visible proximally and dorsal (Additional file 7: Fig. S7F). In the tarantula, *Wnt7.1* expression is restricted to late embryonic stages and mainly in the ventral ectoderm of the appendages, except for the labrum that does not express *Wnt7.1* (Fig. 11A, B; Additional file 4: Figure S4A).

In all spiders, *Wnt7.2* is expressed in the appendages (Figs. 10, 11; Additional files 4, 6, 7: Figs. S4B, S6F, S7G). In *Parasteatoda*, *Wnt7.2* is expressed in the form of several dot-like domains along the dorsum of the labrum, the pedipalps, the legs and the opisthosomal limb buds, but ventral in the chelicerae (Additional file 7: Figure S7G). In addition, there is a dot-like expression ventrally and close to the tip of the legs. In *Pholcus*, however, expression in chelicerae, pedipalps, legs, and opisthosomal appendages is restricted to the dorsal-proximal region (Additional file 6: Figure S6F). In *Acanthoscurria*, expression in the chelicerae is ventral, as described for *Parasteatoda*, and expression in the pedipalps and legs is restricted to a dorsal-proximal patch as described for *Pholcus* (Additional file 4: Figure S4B). Additionally, *Wnt7.2* is expressed in four dominant large domains in the pre-cheliceral region of *Parasteatoda* (Fig. 10E; Additional file 7: Figure S7G). Similar expression is present in *Pholcus* and *Acanthoscurria* albeit in smaller domains (Figs. 10N, 11C). In the spiders *Parasteatoda* and *Pholcus*, *Wnt7.1* and *Wnt7.2*, respectively, are also expressed in the developing ventral nervous system (Fig. 10D, M). In the harvestman *Phalangium*, the single copy of *Wnt7* is only expressed in the dorsal-proximal region of the pedipalps and the legs (but not the labrum or the chelicerae) (Fig. 11F–H; Additional file 5: Figure S5E). Expression of *Wnt7* genes is summarized in schematic Figs. 10O, 11I.

### Wnt8

**Fig. 12:**
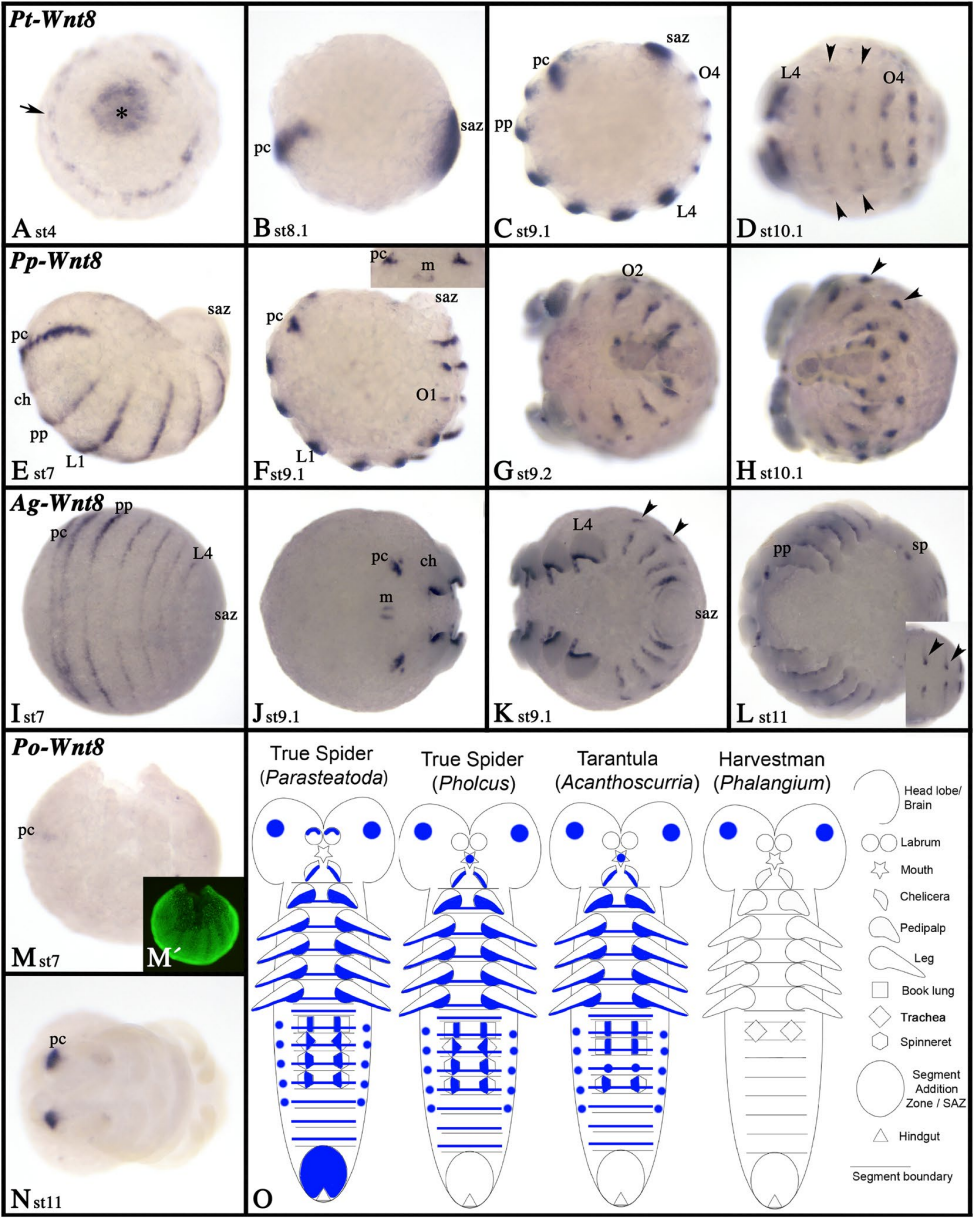
Expression of *Wnt8.* Expression of *Wnt8* in *Parasteatoda* (**A**–**D**), *Pholcus* (**E**–**H**), *Acanthoscurria* (**I**–**L**) and *Phalangium* (**M**, **N**). In all panels, anterior is to the left, except panel **A** where posterior is in the center of the disc (asterisk). Panels **B**, **C**, **F** and **M** show lateral views the other panels represent ventral views. Inlays in panels **F** and **L** show anterior and dorsal aspects respectively. Panel **M´** represents SYBR-green staining of the embryo in **M**. Developmental stages are indicated. Asterisk in **A** marks the center of the germ disc (the later posterior region of the germ band). The arrow in **A** points to expression close to the rim of the germ disc (the later anterior of the germ band). Arrowheads point to dorsal dots of expression in the opisthosoma. Expression patterns are summarized in panel **O**, anterior is up. Abbreviations as in Fig. 4

In all investigated spiders, *Wnt8* is expressed in the ventral ectoderm of the chelicerae, the pedipalps, the legs and the opisthosomal limb buds (Additional files 4, 6, 7: Figs. S4C, S6G, S7H) but only in *Parasteatoda* expression is also present dorsally in the labrum (Additional file 7: Fig. S7H). In *Pholcus* and *Acanthoscurria* (but not *Parasteatoda*), *Wnt8* is expressed in the stomodaeum (Fig. 12F(inlay), J). In all spiders, expression starts early during embryogenesis in the form of transverse segmental stripes that are reminiscent of SPG expression (Fig. 12; Additional file 8: Figure S8). In *Parasteatoda*, expression starts already during the germ disc stage as a central patch and a ring close to the rim of the disc (Fig. 12A). The latter transforms into expression in the pre-cheliceral region, which is also present in the other spiders. The central patch of expression in *Parasteatoda*, however, that later represents expression in the SAZ, is not present in *Pholcus*. Indeed, the earlier reported strong expression of *Wnt8* in the SAZ of *Parasteatoda* [58] is neither present in the entelegyne spider *Pholcus* nor the tarantula *Acanthoscurria*. Like a typical SPG, in all spiders *Wnt8* is expressed in the form of transverse stripes in all newly forming posterior segments (Fig. 12C–F, K; Additional file 8: Fig. 8D). In *Phalangium*, *Wnt8* expression is restricted to two domains in the pre-cheliceral region (Fig. 12M, N). Expression of *Wnt8* is summarized in Fig. 12O.

### Wnt9 and Wnt10

**Fig. 13:**
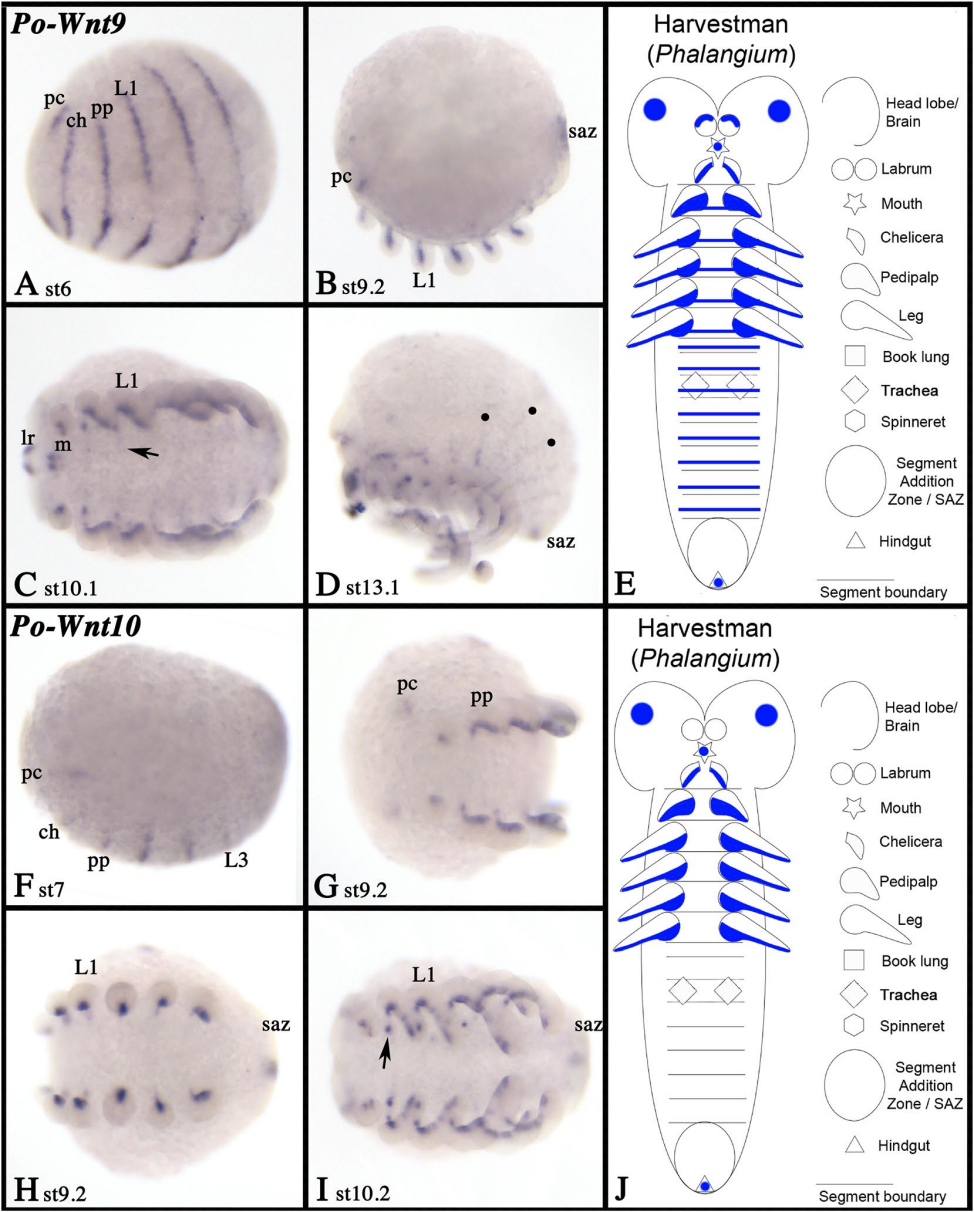
Expression of harvestman *Wnt9* and *Wnt10.* Expression of *Phalangium Wnt9* (**A**–**D**) and *Wnt10* (**F**–**I**). In all panels, anterior is to the left. Panels **A**, **C**, and **G**–**I** represent ventral views. Panels **B**, **D**, and **F** represent lateral views. Developmental stages are indicated. Arrows in panels **C** and **I** point to expression ventral to the base of the appendages. Filled circles in **D** mark dorsal stripes of expression. The arrow in panel **M** points to expression in the endites. Expression patterns of *Wnt9* and *Wnt10* are summarized in panels **E** and **J**, respectively (anterior is up). Abbreviations as in Fig. 4

We did not identify any orthologs of *Wnt9* and *Wnt10* in the spider species studied here. In *Phalangium*, however, we found representatives of both subfamilies (Fig. 3). *Wnt9* is first expressed in a SPG-like pattern as transverse segmental stripes covering the region where the limbs will form and the most ventral tissue of the embryo (Fig. 13A). These early stripes correspond to a domain in the anterior head, the chelicerae-bearing segment, the pedipalpal segment and the first leg-bearing segments. Additional expression is present in the very posterior of the embryo (likely the hindgut primordium) and when posterior segments are added, *Wnt9* is expressed in similar transverse stripes in these segments (Fig. 13B–D). As the appendages develop, expression is restricted to a central sector along the ventral side of the chelicerae, the pedipalps and the legs (and their endites), but in the labrum *Wnt9* is dorsally expressed (Fig. 13B–D; Additional file 5: Fig. S5F). Later during development, expression appears in the stomodaeum (Fig. 13C).

Expression of *Wnt10* also starts early during development and in the form of transverse stripes; note however, that these stripes are not continuous (cf. expression of *Wnt9*). Instead, expression in the ventral region of the embryo is missing (Fig. 13F–I). We assume that these stripes are correlated with the primordia of the appendages. The most anterior expression domains are located in the pre-cheliceral region. Later during development, expression is observed centrally along the ventral side of the appendages (including the endites) (Fig. 13G–I; Additional file 5: Figure S5G). Unlike *Wnt9*, *Wnt10* is not expressed in the labrum. Expression in the posterior pole of the embryo is comparable to that of *Wnt9*, but no stripes were observed in the opisthosomal segments (Fig. 13H, I). Late during embryogenesis, expression of *Wnt10* appears in the stomodaeum (Additional file 5: Figure S5G). Expression of *Wnt9* and *Wnt10* is summarized in Fig. 13E, J, respectively.

### Wnt11

**Fig. 14:**
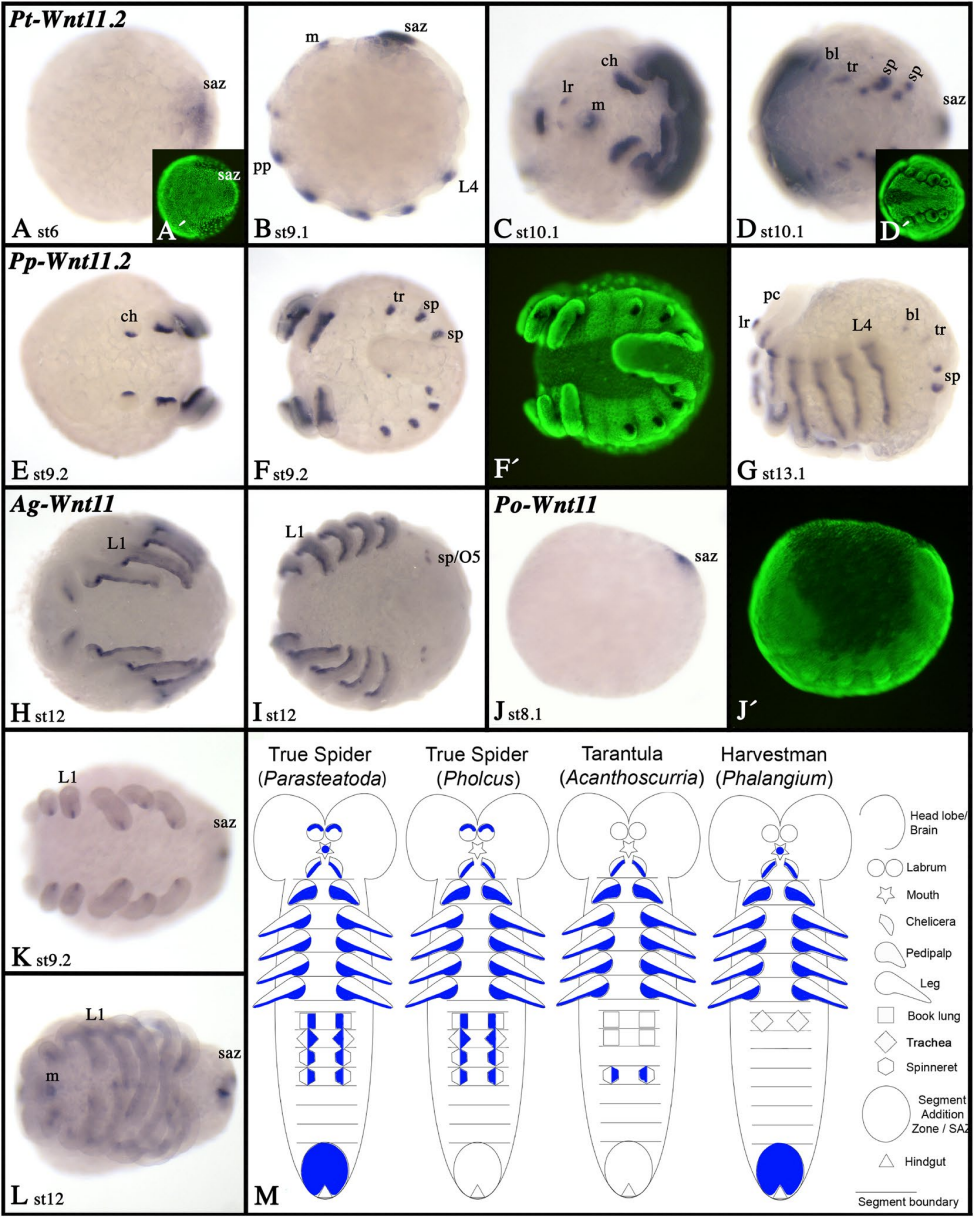
Expression of *Wnt11* genes. Expression of of *Wnt11* in *Parasteatoda* (**A**–**D**), *Pholcus* (**E**–**G**), *Acanthoscurria* (**H**, **I**) and *Phalangium* (**J**–**L**). In all panels, anterior is to the left. Panels **A**, **C**–**F**, **H**, **I**, **K**, and **L** show ventral views. Panels **B**, **G**, and **J** show lateral views. Panels **A´**, **D´**, **F´**, and **J´** represent SYBR-green staining of the embryos shown in corresponding panels. Developmental stages are indicated. Expression patterns are summarized in panel **M**; anterior is up. Abbreviations as in Fig. 4; and tr, trachea

In *Parasteatoda* and *Pholcus*, *Wnt11* is represented by two paralogs (Fig. 3). However, in both species, expression of *Wnt11.1* was not detected in any of the investigated embryonic stages (cf. [39]. In *Acanthoscurria* and *Phalangium* only one copy of *Wnt11* was found. In *Parasteatoda* and *Phalangium*, expression of *Wnt11.2* and *Wnt11*, respectively, appears early during embryogenesis in the SAZ (Fig. 14A, B, J), but in *Pholcus* and *Acanthoscurria*, there is no such posterior expression (Fig. 14F, G, I). In the appendages of all investigated animals (including the opisthosomal buds), expression was observed in the ventral ectoderm, except for the labrum where expression is dorsal (the labrum of the tarantula and the harvestman do not express *Wnt11*) (Fig. 14; Additional files 4, 5, 6, 7: Figs. S4D, S5H, S6H, S7I). Expression in the chelicerae of the harvestman is internal, likely mesodermal (Additional file 5: Fig. S5H). Expression of *Wnt11* is summarized in Fig. 14M.

### Wnt16

**Fig. 15:**
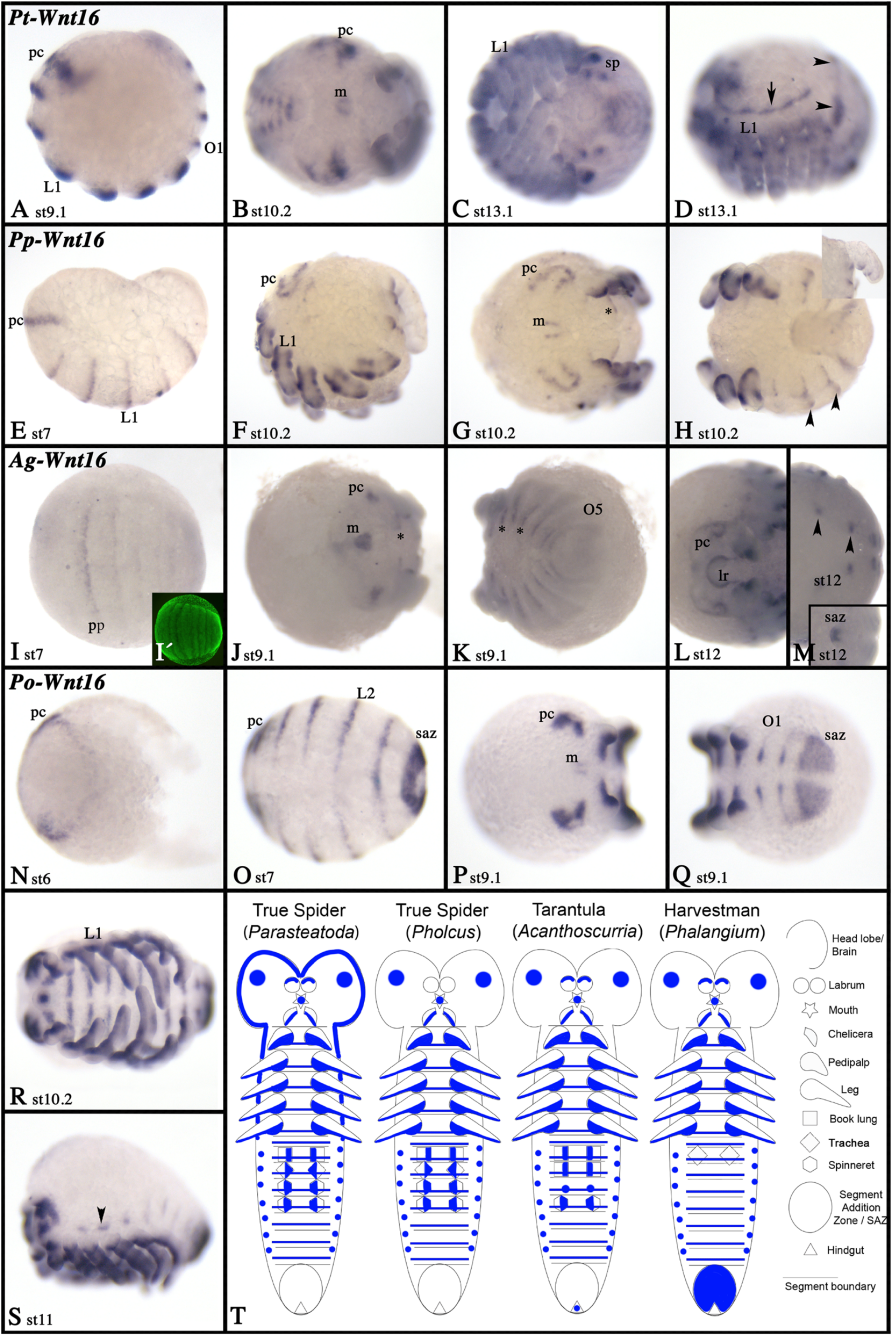
Expression of *Wnt16* genes. Expression of of *Wnt16* in *Parasteatoda* (**A**–**D**), *Pholcus* (**E**–**H**), *Acanthoscurria* (**I**–**M**) and *Phalangium* (**N**–**S**). In all panels, anterior is to the left. Panels **A**, **D**, **E**, **F**, and **S** represent lateral views. Other panels represent ventral views, except panel **M** (dorsal view). The inlay in panel **M** shows the saz of the the same embryo (ventral view). The inlay in H shows a lateral view on the tail and the saz. Panel **I´** represents SYBR-green staining of the embryo shown in I. Arrow and arrowheads in panel **D** point to expression along the dorsal rim of the prosoma and dorsally in the opisthosoma, respectively. Developmental stages are indicated. Arrowheads in panels **H**, **M**, and **S** point to dorsal expression in the opisthosoma. Asterisks in panels **J** and **K** mark expression ventral to the base of the limbs. Expression patterns are summarized in panel **T**; anterior is up. Abbreviations as in Fig. 4

In all investigated species, *Wnt16* is expressed in a SPG-like pattern in the form of transverse segmental stripes (Fig. 15; Additional file 9: Fig. S9). In *Pholcus*, *Acanthoscurria* and *Phalangium*, these stripes appear early during development (cf. Fig. 15E, I, N, O with Additional file 9: Figure S9B), while in *Parasteatoda* the expression starts later coinciding with limb bud formation (Fig. 15A). In spiders, there is no (or only weak) expression in the posterior SAZ, but in the harvestman, *Wnt16* is dominantly expressed in the SAZ (Fig. 15O, Q). In all species, *Wnt16* is also expressed in the pre-cheliceral region and the stomodaeum (Fig. 15A, B, E–G, J, L, P, R, S; Additional files 5, 7: Figs. S5I, S7J). In *Acanthoscurria*, *Wnt16* is expressed on the dorsal side of the labrum and two thin longitudinal stripes of expression run on either side of the stomodaeum (Fig. 15L). Common to all analyzed species, expression in the appendages is restricted to the ventral side including the ventral sector of the endites (if present); in the labrum, expression is always dorsal (Fig. 15; Additional files 4, 5, 6, 7: Figs. S4E, S5I, S6I, S7J). In all spiders, *Wnt16* is expressed in the form of short stripes (or patches) dorsal to the opisthosomal limb buds (Fig. 15D, H, M). Comparable expression is also present in the opisthosomal and the leg-bearing segments in *Phalangium* (Fig. 15S). Expression of *Wnt16* is summarized in Fig. 15T.

### WntA

**Fig. 16:**
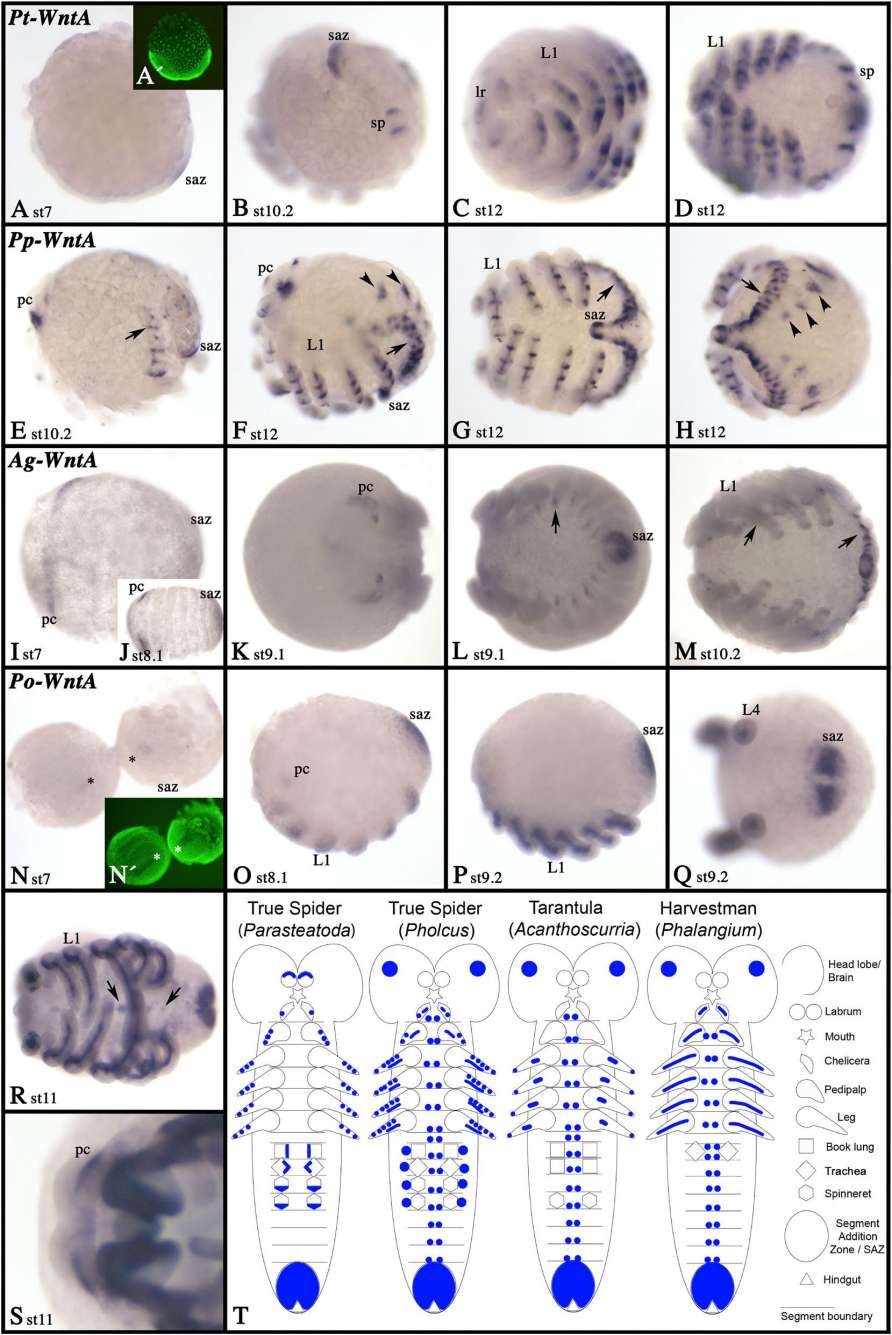
Expression of *WntA* genes. Expression of of *WntA* in *Parasteatoda* (**A**–**D**), *Pholcus* (**E**–**H**), *Acanthoscurria* (**I**–**M**) and *Phalangium* (**N**–**S**). In all panels, anterior is to the left. Panels **A**, **B**, **E**, **F**, **O**, and **P** show lateral views. Panels **C**, **D**, **G**, **I**–**N**, **R**, and **S** represent ventral views. Panels **H** and **Q** show posterior views. Panels **A´** and **N´** represent SYBR-green staining of the embryos shown in corresponding panels. Developmental stages are indicated. Arrowheads in panels **F** and **H** point to expression dorsal to the basis of the opisthosomal appendages. In all panels, arrows point to expression in the ventral nervous system. Expression patterns are summarized in panel **T**; anterior is up. Abbreviations as in Fig. 4

In all species, *WntA* is expressed in the SAZ (Fig. 16). In all species, except *Parasteatoda*, expression is present in the pre-cheliceral region (Fig. 16E, F, K, O, S), and the ventral nervous system along either side of the midline (Fig. 16E–H, L, M, R). Expression in the developing appendages is diverse. In *Phalangium*, expression of *WntA* in chelicerae, pedipalps and legs is exclusively mesodermal.

In *Parasteatoda* and *Pholcus WntA* is expressed in one or several patches in the dorsal ectoderm of the legs, pedipalps and the chelicerae (Additional files 6, 7: Figs. S6J, S7K). Additionally, *WntA* is expressed in the mesoderm of these appendages in *Pholcus* (Additional file 6: Figure S6J). In *Acanthoscurria* expression in the limbs is weak, but still dorsal and distal ectodermal expression domains as well as expression in the mesoderm are present in at least the pedipalps and the legs (Additional file 4: Figure S4F). Only in *Parasteatoda*, *WntA* expression was also observed in the dorsal tissue of the labrum (Fig. 16C; Additional file 7: Figure S7K). Expression of *WntA* is summarized in Fig. 16T.

## Discussion

### Is *Wnt1* (*wingless*) a *bona fide* segment-polarity gene in spiders?

In *Drosophila melanogaster*, the transcription factor encoding gene *engrailed* (*en*) and the signaling molecule encoding gene *Wnt1* (*wingless* (*wg*)) demarcate the parasegmental boundary with *wg* being expressed anterior to this boundary, and *en* being expressed posterior to this boundary (e.g., [17, 26, 63]). Subsequent research in other arthropods and closely related groups like tardigrades and onychophorans revealed that the expression domains of these genes are highly conserved (e.g., [12, 13, 33, 65, 74]).

A deviation from this apparent conservation, however, has previously been suggested for the spider *Parasteatoda* where *Wnt1* is not expressed in the form of a SPG-like pattern or the SAZ [39]. Indeed, already Damen [9] realized that expression of *Wnt1* in the spider *Cupiennius salei* is dissimilar from its expression in other arthropods, and is indeed lacking in cells anterior to *en* in the ventral region of the developing embryo. He suggested that another Wnt gene, *Wnt5*, could perhaps partially substitute the function of *Wnt1* in the ventral tissue, while *Wnt1* would still play its “regular” role as SPG in dorsal tissue [9]. Although this appears to be an interesting idea, a closer look at the expression of *Wnt5* in *Cupiennius* and other chelicerates reveals a likely role in the patterning of the ventral nervous system, rather than a role as a SPG (Fig. 8). Although *Wnt5* is expressed relatively early during embryogenesis in arthropods, and the initial expression in the early germ band is in the form of transverse stripes, these stripes soon transform into patch-like domains in the ventral nervous system, and the domains in posteriorly added segments never develop into SPG-like stripes [9, 39], this study). Consequently, *Wnt5* likely does not act in combination with *Wnt1* during spider segmentation. Both papers, Damen [9] and Janssen et al. [39] also suggested that a second *Wnt1* gene could exist in spiders that could pattern the ventral tissue. It was therefore exciting to discover two *Wnt1* paralogs in the spider *Acanthoscurria*, but neither of the *Wnt1* genes in this species is expressed like a SPG (Fig. 4). In *Pholcus*, *Wnt1* is also not expressed like a typical SPG, but instead (as with *Parasteatoda*) is only detected in the form of transverse stripes in a subset of the anterior segments, and no such stripes appear in the newly forming posterior segments (Fig. 4). It appears thus that at least in spiders, *Wnt1* does not function as a *bona fide* SPG. In the harvestman, however, *Wnt1* is expressed in the form of a typical SPG, and hence it is likely that in this group of arachnids, the ancestral function of *Wnt1* has been retained (Fig. 5). It would be interesting to analyze the expression of *Wnt1* genes in other Arachnopulmonata, especially whip spiders which also appear to have retained two copies of this gene after the ancestral WGD [21] to better understand the evolution of this gene in chelicerates.

**Fig. 17:**
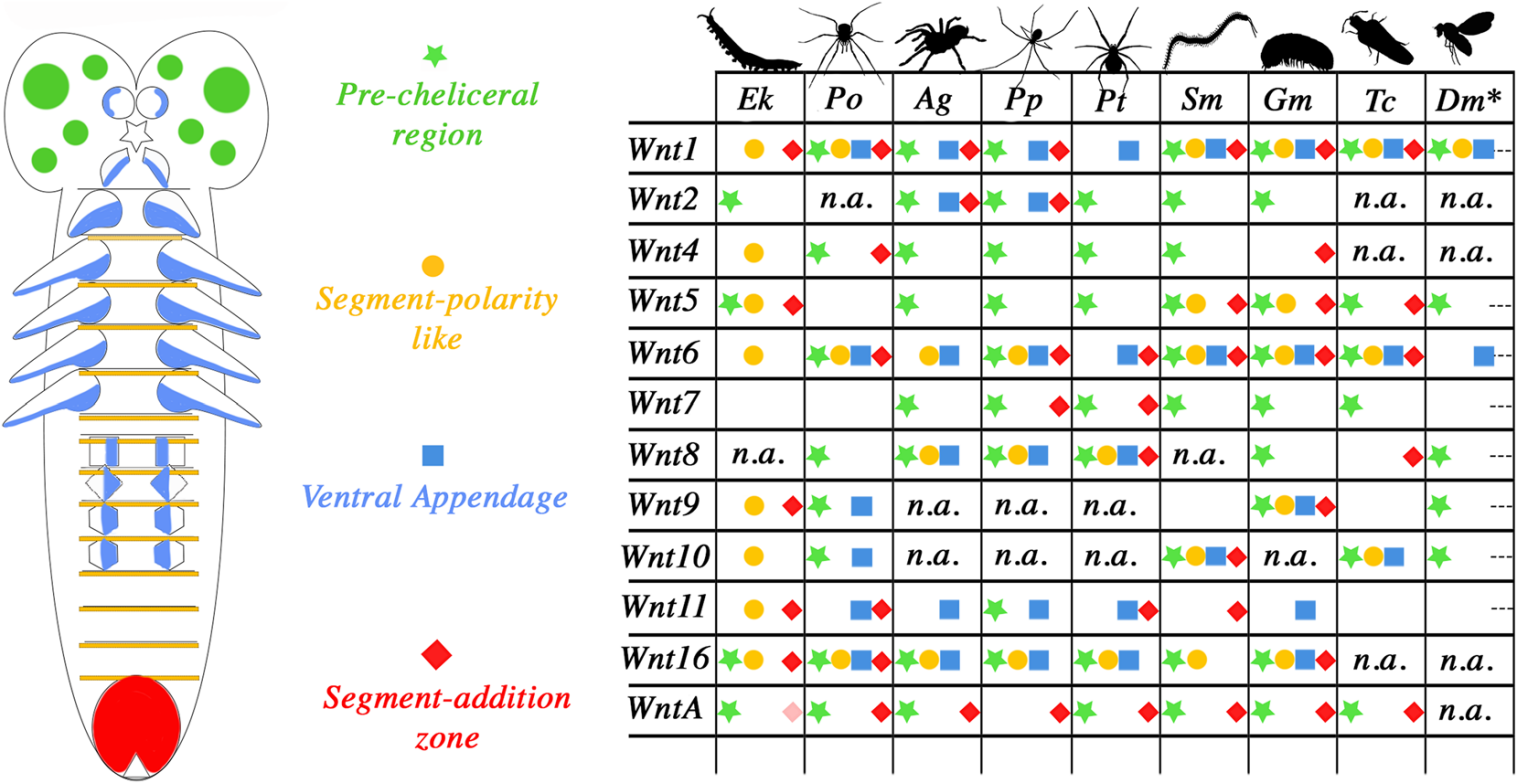
Schematic overview of conserved aspect of Wnt gene expression. Green expression (and green stars) represents expression in the pre-cheliceral region. Blue expression (and blue squares) represents expression in the ventral ectoderm of the appendages. Yellow expression (and yellow dots) represents segment-polarity gene like expression. Red expression (and red diamond) represents expression in the segment-addition zone. Note that data from *Drosophila* imaginal discs are often missing (*). The pale red diamond in *Ek*/*WntA* represents weak expression. In general, data-completeness may suffer from non-comprehensive studies. Data are mainly based on Hogvall et al. [28], Hayden and Arthur [22], Janssen et al. [39], Janssen and Posnien [36], Bolognesi et al. [4], Murat et al. [62] (and references therein), and this study. Species abbreviations: *Ek*, *Euperipatoides kanangrensis* (Onychophora), *Po*, *Phalangium opilio* (Chelicerata: Opiliones), *Ag*, *Acanthoscurria geniculata* (Chelicerata: Mygalomorpha), *Pp*, *Pholcus phalangioides* (Chelicerata: Aranea), *Pt*, *Parasteatoda tepidariorum* (Chelicerata: Aranea); *Sm*, *Strigamia maritima* (Myriapoda: Chilopoda); *Gm*, *Glomeris marginata* (Myriapoda: Diplopoda); *Tc*, *Tribolium castaneum* (Hexapoda: Coleoptera); *Dm*, *Drosophila melanogaster* (Hexapoda: Diptera)

Could another Wnt gene substitute for *Wnt1*-function during segmentation in spiders? Our analysis shows that several Wnt genes are indeed expressed in a pattern that is similar to the expression of *Wnt1* in other arthropods (summarized in Fig. 17). Besides the expression in the form of transverse segmental stripes anterior to *en* (i.e., in about the middle of the segment), another important factor is the temporal appearance of expression: a substitute for *Wnt1* should be expressed early during segment formation.

In *Pholcus*, *Acanthoscurria* and even in *Phalangium* (except for *Wnt8*), *Wnt6*, *Wnt8*, and *Wnt16* are expressed like SPGs during segment formation in both the anterior segments that form from the early blastoderm and the germ disc, and the posterior segments that are added from the posterior SAZ (Figs 9, 12, 15, 17; Additional files 8, 9: Figs. S8, S9). In *Parasteatoda*, however, *Wnt6* is not expressed in a SPG-like fashion (Fig. 9), and *Wnt8* has been extensively studied, and it has been found that it is an important factor for the establishment of the SAZ and thus posterior elongation [58]. Although expressed in the germ disc (from which the anterior segments are formed) there are no obvious anterior SPG-like phenotypes in *Wnt8* knock-down embryos [58]. However, a SPG-like function could be masked by the function of yet another Wnt gene such as *Wnt16*. If *Wnt8* substitutes partially for *Wnt1*, then this function may have evolved in the lineage leading to spiders (or any lineage within Arachnopulmonata), because in the outgroup, the harvestman *Phalangium*, *Wnt8* is not expressed in a SPG-like pattern. In arthropods outside Chelicerata, *Wnt8* genes are either missing, or their expression (and function) is quite diverse [4, 5, 14, 16, 22, 30, 39]. This suggests that *Wnt8* has flexibility to assume different functions during evolution, and this may speak for *Wnt8* as a potential (at least partial) substitute for *Wnt1* in spiders (Fig. 17).

The most likely candidate for substituting for *Wnt1* function in spider segmentation, however, appears to be *Wnt16*. In all chelicerate species, *Wnt16* is expressed in a typical SPG-like pattern both during anterior and posterior segment formation (Fig. 15; Additional file 9: Fig. S9). In other arthropods, and even in an onychophoran, *Wnt16* is also expressed in a SPG-like pattern suggesting a conserved role in segmentation [8, 22, 28, 39]. *Wnt16* has thus far not been in the focus of scientific studies, and this may be correlated to the fact that holometabolous insects, the most intensively studied arthropod species (cf. data on *Drosophila* (reviewed in Murat et al. [62], have lost *Wnt16* (e.g., [39], [21]. In this context, it would be interesting to investigate the expression of *Wnt16* in insects that have retained this gene, and to perform *Wnt16* knock-down studies in spiders.

### Wnt-signaling is likely involved in posterior elongation, but *Wnt8* is not a conserved factor in this network

*Wnt8* is one of the few arthropod Wnt genes for which functional data exist outside *Drosophila*. In the spider *Parasteatoda* and the beetle *Tribolium castaneum*, RNAi-mediated knockdown of *Wnt8* results in truncated embryos. This has been interpreted as evidence that *Wnt8* represents a conserved component of an ancestral posterior gene regulatory network in arthropods [5, 58, 79], or even in animals in general (e.g., [47, 49], reviewed in [59]). In many arthropods, however, *Wnt8* has been lost (e.g., [21]). In such species, another Wnt gene must regulate posterior segment addition, as exemplified for the cockroach *Periplaneta americana*, where knockdown of *Wnt1* causes posterior truncation [6]. This is not unexpected because Wnt-patterning likely includes a high degree of redundancy and combinatorial gene function as suggested by the similar expression patterns of multiple different Wnt genes in any given species (e.g., [4, 8, 22, 39]), and as shown for *Wnt1* and *Wnt8* in *Tribolium* [5].

In the spiders we studied here, *Wnt8* is not expressed at the posterior pole of the embryo with the exception of *Parasteatoda* (Figs. 12, 17). The most parsimonious explanation is thus that the role of *Wnt8* in *Parasteatoda* represents an apomorphy for this spider species, or possibly Entelegynae as a whole, but not for spiders or chelicerates in general; note that *Wnt8* is not expressed in the SAZ in the harvestman *Phalangium* either. Similarly, the posterior expression of *Wnt8* in *Tribolium* may represent a synapomorphy of *Tribolium* or beetles in general because *Wnt8* is missing or not expressed in the SAZ of other arthropods such as myriapods and crustaceans and other insects (e.g., [10, 39]). This finding further strengthens the view that Wnt genes can be co-opted into existing gene regulatory networks to work in combination with or even replace the function of another Wnt gene.

### Wnt-signaling in anterior–posterior axis elongation

In all previously investigated species that develop via posterior elongation, which is the vast majority of all arthropods, and also the vast majority of animals in general, at least one Wnt ligand is always expressed posteriorly in the developing embryo, and loss of one or more Wnt genes causes truncation of the main body axis (reviewed in [53, 93]). Equally, knocking-down the function of Wnt-signaling by targeting key-components of Wnt pathways, or inducing over-activity of Wnt-signaling, lead to posteriorly truncated embryos or disturbances in the posterior patterning network (e.g., [2, 23, 75, 82])

Is there an “ancestral” posterior Wnt factor? In *Tribolium*, double knockdown of *Wnt1* + *Wnt8* causes more severe effects than the mere knockdown of either of these two genes alone suggesting that they may work together [5]. In another species, the cockroach *Periplaneta*, knockdown of *Wnt1* also results in truncated embryos, further suggesting that *Wnt1* may be an ancestral factor of posterior elongation, at least in insects [6]. Data from the cricket *Gryllus bimaculatus* and the true bug *Oncopeltus fasciatus*, however, show that knockdown of *Wnt1* has no effect on posterior elongation, although disruption of the complete canonical Wnt pathway causes truncation suggesting that *Wnt1* may act in combination with other Wnt factors [1, 61], reviewed in [93]. Interestingly, however, *Wnt1* cannot be involved in posterior elongation in *Parasteatoda* because it is not expressed in the posterior of the embryo [39] (Fig. 4). However, *Wnt1* shows posterior embryonic expression in most arthropod species and in outgroups such as onychophorans and priapulids (e.g., [12, 54]) (Fig. 17). *Wnt1* is thus likely a conserved factor of posterior elongation, and the situation in the model spider *Parasteatoda* likely presents a derived feature.

To further investigate the possibility that other Wnt genes may be involved in posterior elongation we summarized the findings from arthropods, an onychophoran and a priapulid, all for which comprehensive expression data of the complete complement of Wnt genes are available [4, 8, 22, 28, 29, 36, 39, 40]. Several Wnt genes are typically expressed in the posterior embryo, but often their distribution is little conserved among different species including arthropods. These genes could, however, still contribute to posterior elongation and segment addition, either alone or in concert with other Wnt genes (Fig. 17). However, the summary of posteriorly expressed *Wnt* genes reveals two other *Wnt* genes beyond *Wnt1* that are expressed in the posterior of developing embryos of most species. *Wnt6* is expressed posteriorly in the priapulid and all arthropods except *Acanthoscurria* (Fig. 17) [29]*.* Like many Wnt genes, *Wnt6* is highly under-investigated and so expression data are relatively scarce and the function of this gene has not been studied in many species. Interestingly, however, *Wnt1* and *Wnt6* appear to be ancient paralogs as revealed by phylogenetic analyses (e.g., [7, 10, 21, 29, 39], this study) and their conserved synteny in at least insects and crustaceans (data on Wnt gene synteny in other arthropods are not available), a lophotrochozoan species, the owl limpet *Lottia* [10, 39], and some chordates [84]. In addition, *Wnt1* and *Wnt6* have overlapping expression patterns in many species (e.g., [4, 35, 39], this study). It is therefore possible that *Wnt6* may have had an ancestral role in posterior elongation like *Wnt1*. To test this further the function of *Wnt6* should be assessed via gene knockdown in species where this technique is established and where *Wnt6* is expressed in the posterior of the embryo, including the beetle *Tribolium* [4].

Another Wnt gene with posterior expression in all investigated arthropod species, and even the onychophoran (albeit weakly) and the priapulid, is *WntA* (Figs. 16, 17) [29]. In *Tribolium*, knockdown of *WntA* does not cause any phenotype, neither on its own nor in combination with *Wnt1* and/or *Wnt8* [5]. Although *WntA* is thus likely not involved in posterior segmentation in *Tribolium*, this does not exclude the possibility that it is in other arthropods. In order to answer this question conclusively, further research is required including functional studies.

### Wnt genes in arthropod appendage development

In *Drosophila*, Wnt-signaling is an important regulator of limb development. In the developing limb discs, *Wnt1* (*wg*) is expressed in the ventral sector of the disc, and loss of its function causes dorsalization of the limbs. In the dorsal sector of the discs, *decapentaplegic* (*dpp*) and its downstream target gene *optomotor-blind* (*omb*) are expressed (reviewed in [70]). In all hitherto investigated arthropods, the expression of *Wnt1* and *omb* during limb development are highly conserved suggesting that their function is conserved as well (e.g., [38, 71, 72], this study). In *Tribolium*, functional studies revealed conserved function of *Wnt1* in ventral limb development [18]. A functional study in a hemimetabolous insect, the true bug *Oncopeltus*, however, suggested that this function may be restricted to holometabolous insects [1]. Other functional data on the possible function of *Wnt1* in limb development are not available, and it is therefore unclear if the situation in *Oncopeltus* is conserved in other arthropods, or if it represents an exception. In any case, a reoccurring feature of Wnt genes is their expression along the ventral side of outgrowing appendages (Fig. 17). Expression of Wnt genes in the dorsal of appendages, however, is much rarer and never in the same striking continuous patterns as displayed for the ventral side (except for the labrum that likely rotated by 180° during evolution and therefore expresses Wnt genes predominantly along its dorsal side [45]. In onychophorans, however, a closely related group of animals, Wnt genes are expressed in the tips of the growing appendages [12, 28]. Thus, the ventral appendage-patterning by the Wnt genes might represent a conserved feature restricted to arthropods. Either way, the fact that multiple Wnts are expressed along the ventral side of the developing appendages in all investigated arthropod species strongly suggest that they have a function in ventral limb development, either individually or in combination. Therefore, functional studies targeting a single Wnt gene, as performed in *Oncopeltus* [1], could easily overlook the involvement of Wnt-patterning in ventral vs dorsal appendage development. To circumvent problems caused by redundant function(s) of multiple Wnts in studying arthropod limb development, known downstream targets of Wnt, such as the T-box encoding transcription factor *H15*/*midline,* could instead be addressed by means of e.g., RNAi-mediated knockdown [38, 71, 86, 87]. Another transcription factor that is expressed along the ventral sector of all appendages in all arthropods and even an onychophoran is the forkhead-box encoding gene *FoxB*. This gene appears to act upstream of Wnt-signaling and may thus provide yet another alternative to study the role(s) of Wnt-signaling in appendage development [24].

### Insight into the complexity of arthropod Wnt-patterning: a potpourri of functional redundancy, combinatorial function, function-shuffling, and neo- and sub-functionalization

Wnt-patterning, the interaction of the multiple Wnt ligands with the plentitude of their potential receptors, is highly complex (e.g., [25, 32, 50]). We can assume that many (if not the most) Wnts possess very similar biochemical features, such as their receptor-binding sites (e.g., [34, 77]. As a consequence, Wnts are in many cases able to interact with more than one type of receptor, and multiple Wnts can likely interact (albeit with different stringency) with the same receptor [43]. As a result, a given Wnt can be co-opted relatively easy into a GRN replacing another Wnt (e.g., [55]. For the same reasons, different Wnts can act redundantly, as long as they share the same regulatory elements and are thus co-expressed. Co-expression also allows Wnt genes to function combinatorial (e.g., [4, 5, 39, 91]). The control of a given developmental feature or genetic interaction can thus be under control of a set of Wnt genes, possibly in a dose-dependent manner, (reviewed in e.g., [94]), rather than a single Wnt. In summary, this provides a complex network of mutational protection, and thus the loss of one of these redundant/complementary Wnt factors (caused by either depletion of the gene, or regulatory changes) may not alter the development of the organism very much. Indeed, it has been shown that function-shuffling occurs regularly in Wnt genes, often associated with gene loss [52, 56, 84]. The latter, however, is not mandatory, especially when the gene is part of multiple GRNs. In spiders, we frequently observe Wnt gene expression domain losses and gains, such as the dominant posterior expression of *Wnt8* in *Parasteatoda*, but in no other spider, or the loss of the segment-polarity like pattern of *wg/Wnt1* in spiders, although this pattern is conserved in the harvestman and arthropods in general (both cases discussed above). Gain of an expression pattern on the other hand is for example represented by the expression of *Wnt2* in the SAZ and the ventral surface of the appendages in basally branching spiders (possibly followed by a loss in entelegyne spiders) (summarized in Fig. 17). Although function-shuffling is not necessarily accompanied by gene loss, it could explain the loss of *Wnt9* and *Wnt10* class genes in spiders (Fig. 3). Function-shuffling could also explain why Wnt genes are often expressed in similar patterns, e.g., along the ventral side of the appendages, a feature that cannot easily be explained by ancestry. The reconstruction of the ancestral patterns of Wnt genes is also likely impeded by function-shuffling (associated with the acquirement of shared expression patterns). The reoccurring expression of Wnts in the SAZ (likely associated with posterior elongation) and the regionalization of the brain could represent ancestral features of Wnt gene function because the central nervous system and posterior elongation are ancestral features of most animals. Reoccurring expression along the ventral side of the appendages and the segment-polarity like patterns, however, likely are conserved features of (pan)arthropods and thus must have evolved in the lineage leading to this group of animals, long after the establishment of the protostomian Wnt complement (e.g., [39]).

Another feature observed for Wnt genes is the retention of both copies after duplication that adds yet another level of complexity. As we see in spiders, duplicated genes always display quite different expression pattern, suggesting that these genes have not been incorporated into the redundancy-based mutational protection network that the complexity of Wnt gene expression most likely provides. Instead, if retained, one copy of a given Wnt gene must have required new functions and thus expression patterns (neo-functionalization) (e.g., *Wnt4* and *Wnt7*, Figs. 7, 10 and 11). Compared to other genes, most copies of Wnt genes disappeared after duplication (cf. with duplicated and almost fully retained Hox gene clusters in spiders (e.g., [80, 81] or the multitude of duplicated homeodomain genes [46]). This further strengthens the idea that the interaction of Wnt genes is dose-dependent and may be disturbed by the presence and transcription of a new duplicate. Cases of sub-functionalization, i.e., the subdivision of function and thus expression are rather rare in duplicated Wnt genes. One impressive example, however, is represented by the expression of the two *Wnt1* ohnologs in the tarantula *Acanthoscurria* (Fig. 4).

The fact that many Wnt genes are expressed in similar patterns demands comprehensive studies including all genes that share a given expression pattern in order to investigate the function of “Wnt” in a given developmental or evolutionary context. As this study shows and tries to highlight, these Wnt genes may not necessarily be paralogs, but may represent members of other classes of Wnt genes. As the expression of Wnt genes appears to change frequently during the course of evolution, possibly as a result of function-shuffling or the general exchange of regulatory elements, developmental studies concerning the function of a given Wnt gene should rather address Wnt gene patterning as a whole (the complement of Wnt genes with identical/similar expression). Future evolutionary studies, comparing of gene expression and their function among a variety of more or less related animals, however, should include a sufficient number of species along the phylogenetic tree to reveal possibly changing expression patterns (and potential function). The latter is of the uttermost importance in order to draw any relevant conclusion from such data in terms of evolutionary processes. Essential to both kinds of studies is the comprehensive knowledge about Wnt gene expression in any given research organism, a task this paper aims to contribute to.

## Supplementary Information

**Additional file 1:** **Fig. S1** The complements of arthropod and onychophoran Wnt genes. Full species names that are not listed in the legend of Fig. 1: *Acyrthosiphon pisum* (Hexapoda: Homoptera), *Anopheles gambiae* (Hexapoda: Diptera); *Apis mellifera* (Hexapoda: Hymenoptera); *Daphnia pulex* (“Crustacea”: Branchiopoda); *Drosophila melanogaster* (Hexapoda: Diptera); *Euperipatoides kanangrensis* (Onychophora); *Glomeris marginata* (Myriapoda: Diplopoda); *Strigamia maritima* (Myriapoda: Chilopoda), *Thamnocephalus platyurus* (“Crustacea”: Branchiopoda); *Tribolium castaneum* (Hexapoda: Coleoptera). Abbreviations: e, expression has been studied, but no specific signal has been reported; E, expression has been studied; F, functional studies have been performed.

**Additional file 2:** **Fig. S2**. Early expression of *Phalangium Wnt1.* A Posterior view, anterior to the left. B Dorsal view, anterior to the left. C and D, posterior views, anterior to the left. Developmental stages are indicated. The asterisks mark the posterior of the embryo proper. Abbreviations: df, dorsal field; saz, segment-addition zone.

**Additional file 3:** **Fig. S3**. Expression in the appendages of *Acanthoscurria.* Abbreviations: (l), lateral view; p (posterior view); ch, chelicera; en, endite; L, leg; pp, pedipalp. Appendage-type and orientation are the same for all Wnt genes, as indicated for *Wnt1.1*.

**Additional file 4:** **Fig. S4**. Expression in the appendages of *Acanthoscurria* (continued). Abbreviations: (l), lateral view; p (posterior view); ch, chelicera; en, endite; L, leg; pp, pedipalp. Appendage-type and orientation are the same for all Wnt genes, as indicated for *Wnt7.1*.

**Additional file 5:** **Fig. S5** Expression in the appendages of *Phalangium.* All panels show anterior views. Appendage-type and orientation are the same for all Wnt genes, as indicated for *Wnt1*. The asterisk marks the tip of the chelicerae that often attract unspecific staining at late developmental stages. Abbreviations: ch, chelicera; en, endite; L, leg; lr, labrum; m, mouth; pc, pre-cheliceral region; pp, pedipalp.

**Additional file 6:** **Fig. S6**. Expression in the appendages of *Pholcus.* All appendages are shown from ventral, except last panel in I (posterior view of a leg). Appendage-type and orientation are the same for all Wnt genes, as indicated for *Wnt1*. Abbreviations: ch, chelicera; L, leg; pp, pedipalp.

**Additional file 7:** **Fig. S7**. Expression in the appendages of *Parasteatoda.* Labrum and chelicerae are shown from anterior, pedipalps and legs are shown from ventral. Appendage-type and orientation are the same for all Wnt genes, as indicated for *Wnt1*. Arrows point to expression in the labrum. Asterisks mark expression at the doral rim of the head. Abbreviations: ch, chelicera; en, endite; L, leg; m, mouth; pc, pre-cheliceral region; pp, pedipalp.

**Additional file 8:** **Fig. S8.** Early expression of *Parasteatoda* and *Acanthoscurria Wnt8.* In all panels, anterior is to the left, ventral views (except panels A and C (lateral views)). Developmental stages are indicated. Panels marked with an apostrophe represent SYBR-green images of the embryo shown in the regular panels. Abbreviations as in Fig. 4.

**Additional file 9:** **Fig. S9.** Early expression of Parasteatoda *Wnt16.* In all panels, anterior is to the left, ventral views (except panel B (lateral view)). Inlay picture in A represents SYBR-green image of the embryo shown in the regular panel. Developmental stages are indicated. Abbreviations as in Fig. 4.

**Additional file 10.** Wnt gene alignment.

**Additional file 11.** Accession Numbers.

**Additional file 12.** Primers.

## Data Availability

All data generated or analyzed during this study are included in this article (and its additional information files).

## References

[1] Angelini DR, Kaufman TC (2005). Functional analyses in the milkweed bug *Oncopeltus fasciatus* (Hemiptera) support a role for Wnt signaling in body segmentation but not appendage development. Dev Biol.

[2] Beermann A, Prühs R, Lutz R, Schröder R (2011). A context-dependent combination of Wnt receptors controls axis elongation and leg development in a short germ insect. Development.

[3] Bolger AM, Lohse M, Usadel B (2014). Trimmomatic: a flexible trimmer for Illumina sequence data. Bioinformatics.

[4] Bolognesi R, Beermann A, Farzana L, Wittkopp N, Lutz R, Balavoine G, Brown SJ, Schröder R (2008). Tribolium Wnts: evidence for a larger repertoire in insects with overlapping expression patterns that suggest multiple redundant functions in embryogenesis. Dev Genes Evol.

[5] Bolognesi R, Farzana L, Fischer TD, Brown SJ (2008). Multiple Wnt genes are required for segmentation in the short-germ embryo of *Tribolium castaneum*. Curr Biol.

[6] Chesebro JE, Pueyo JI, Couso JP (2013). Interplay between a Wnt-dependent organiser and the Notch segmentation clock regulates posterior development in *Periplaneta americana*. Biol Open.

[7] Cho SJ, Vallès Y, Giani VC, Seaver EC, Weisblat DA (2010). Evolutionary dynamics of the wnt gene family: a lophotrochozoan perspective. Mol Biol Evol.

[8] Constantinou SJ, Pace RM, Stangl AJ, Nagy LM, Williams TA (2016). Wnt repertoire and developmental expression patterns in the crustacean *Thamnocephalus platyurus*. Evol Dev.

[9] Damen WG (2002). Parasegmental organization of the spider embryo implies that the parasegment is an evolutionary conserved entity in arthropod embryogenesis. Development.

[10] Ding X, Liu J, Zheng L, Song J, Li N, Hu H, Tong X, Dai F (2019). Genome-wide identification and expression profiling of Wnt family genes in the Silkworm, *Bombyx mori*. Int J Mol Sci.

[11] Dunlop JA, Lamsdell JC (2017). Segmentation and tagmosis in Chelicerata. Arthropod Struct Dev.

[12] Eriksson BJ, Tait NN, Budd GE, Akam M (2009). The involvement of engrailed and wingless during segmentation in the onychophoran *Euperipatoides kanangrensis* (Peripatopsidae: Onychophora) (Reid 1996). Dev Genes Evol.

[13] Gabriel WN, Goldstein B (2007). Segmental expression of Pax3/7 and engrailed homologs in tardigrade development. Dev Genes Evol.

[14] Ganguly A, Jiang J, Ip YT (2005). Drosophila WntD is a target and an inhibitor of the Dorsal/Twist/Snail network in the gastrulating embryo. Development.

[15] Garwood RJ, Dunlop JA, Selden PA, Spencer AR, Atwood RC, Vo NT, Drakopoulos M (2016). Almost a spider: a 305-million-year-old fossil arachnid and spider origins. Proc Biol Sci.

[16] Gordon MD, Dionne MS, Schneider DS, Nusse R (2005). WntD is a feedback inhibitor of Dorsal/NF-kappaB in Drosophila development and immunity. Nature.

[17] Gritzan U, Hatini V, DiNardo S (1999). Mutual antagonism between signals secreted by adjacent wingless and engrailed cells leads to specification of complementary regions of the Drosophila parasegment. Development.

[18] Grossmann D, Scholten J, Prpic NM (2009). Separable functions of wingless in distal and ventral patterning of the Tribolium leg. Dev Genes Evol.

[19] Haas BJ, Papanicolaou A, Yassour M, Grabherr M, Blood PD, Bowden J, Couger MB, Eccles D, Li B, Lieber M, MacManes MD, Ott M, Orvis J, Pochet N, Strozzi F, Weeks N, Westerman R, William T, Dewey CN, Henschel R, LeDuc RD, Friedman N, Regev A (2013). De novo transcript sequence reconstruction from RNA-seq using the Trinity platform for reference generation and analysis. Nat Protoc.

[20] Harmer AM, Blackledge TA, Madin JS, Herberstein ME (2011). High-performance spider webs: integrating biomechanics, ecology and behaviour. J R Soc Interface.

[21] Harper A, Baudouin-Gonzalez L, Schönauer A, Janssen R, Seiter M, Holzem M, Arif S, McGregor AP, Sumner-Rooney L. Widespread retention of ohnologs in key developmental gene families following whole genome duplication in arachnopulmonates. bioRxiv 2021. 07.10.177725. 10.1093/g3journal/jkab299 PMC8664421 34849767

[22] Hayden L, Arthur W (2014). The centipede Strigamia maritima possesses a large complement of Wnt genes with diverse expression patterns. Evol Dev.

[23] Hayden L, Schlosser G, Arthur W (2015). Functional analysis of centipede development supports roles for Wnt genes in posterior development and segment generation. Evol Dev.

[24] Heingård M, Turetzek N, Prpic NM, Janssen R (2019). FoxB, a new and highly conserved key factor in arthropod dorsal-ventral (DV) limb patterning. EvoDevo.

[25] Hendriks B, Reichmann E (2002). Wnt signaling: a complex issue. Biol Res.

[26] Hidalgo A (1991). Interactions between segment polarity genes and the generation of the segmental pattern in Drosophila. Mech Dev.

[27] Hilbrant M, Damen WG, McGregor AP (2012). Evolutionary crossroads in developmental biology: the spider *Parasteatoda tepidariorum*. Development.

[28] Hogvall M, Schönauer A, Budd GE, McGregor AP, Posnien N, Janssen R (2014). Analysis of the Wnt gene repertoire in an onychophoran provides new insights into the evolution of segmentation. EvoDevo.

[29] Hogvall M, Vellutini BC, Martín-Durán JM, Hejnol A, Budd GE, Janssen R (2019). Embryonic expression of priapulid Wnt genes. Dev Genes Evol.

[30] Holzem M, Braak N, Brattström O, McGregor AP, Breuker CJ. Wnt gene expression during early embryogenesis in the nymphalid butterfly *Bicyclus anynana*. Front Ecol Evol. 2019:7.

[31] Huelsenbeck JP, Ronquist F (2001). MRBAYES: bayesian inference of phylogenetic trees. Bioinformatics.

[32] Huelsken J, Behrens J (2002). The Wnt signalling pathway. J Cell Sci.

[33] Hughes CL, Kaufman TC (2002). Exploring myriapod segmentation: the expression patterns of even-skipped, engrailed, and wingless in a centipede. Dev Biol.

[34] Janda CY, Waghray D, Levin AM, Thomas C, Garcia KC (2012). Structural basis of Wnt recognition by Frizzled. Science.

[35] Janson K, Cohen ED, Wilder EL (2001). Expression of DWnt6, DWnt10, and DFz4 during Drosophila development. Mech Dev.

[36] Janssen R, Prpic NM, Damen WG (2004). Gene expression suggests decoupled dorsal and ventral segmentation in the millipede *Glomeris marginata* (Myriapoda: Diplopoda). Dev Biol.

[37] Janssen R, Damen WG (2008). Diverged and conserved aspects of heart formation in a spider. Evol Dev.

[38] Janssen R, Feitosa NM, Damen WG, Prpic NM (2008). The T-box genes H15 and optomotor-blind in the spiders *Cupiennius salei*, *Tegenaria atrica* and *Achaearanea tepidariorum* and the dorsoventral axis of arthropod appendages. Evol Dev.

[39] Janssen R, Le Gouar M, Pechmann M, Poulin F, Bolognesi R, Schwager EE, Hopfen C, Colbourne JK, Budd GE, Brown SJ, Prpic NM, Kosiol C, Vervoort M, Damen WG, Balavoine G, McGregor AP (2010). Conservation, loss, and redeployment of Wnt ligands in protostomes: implications for understanding the evolution of segment formation. BMC Evol Biol.

[40] Janssen R, Posnien N (2014). Identification and embryonic expression of Wnt2, Wnt4, Wnt5 and Wnt9 in the millipede Glomeris marginata (Myriapoda: Diplopoda). Gene Expr Patterns.

[41] Janssen R, Schönauer A, Weber M, Turetzek N, Hogvall M, Goss GE, Patel NH, McGregor AP, Hilbrant M (2015). The evolution and expression of panarthropod frizzled genes. Front Ecol Evol.

[42] Janssen R, Andersson E, Betnér E, Bijl S, Fowler W, Höök L, Leyhr J, Mannelqvist A, Panara V, Smith K, Tiemann S (2018). Embryonic expression patterns and phylogenetic analysis of panarthropod sox genes: insight into nervous system development, segmentation and gonadogenesis. BMC Evol Biol.

[43] Jiang X, Cong F (2016). Novel regulation of Wnt signaling at the proximal membrane level. Trends Biochem Sci.

[44] Juberthie C. Recherches sur la biologie des Opilions. Dissertation, Universit´e de Toulouse, Toulouse, France. 1964.

[45] Kimm MA, Prpic NM (2006). Formation of the arthropod labrum by fusion of paired and rotated limb-bud-like primordia. Zoomorphology.

[46] Leite DJ, Baudouin-Gonzalez L, Iwasaki-Yokozawa S, Lozano-Fernandez J, Turetzek N, Akiyama-Oda Y, Prpic NM, Pisani D, Oda H, Sharma PP, McGregor AP (2018). Homeobox gene duplication and divergence in Arachnids. Mol Biol Evol.

[47] Li HY, Bourdelas A, Carron C, Gomez C, Boucaut JC, Shi DL (2006). FGF8, Wnt8 and Myf5 are target genes of Tbx6 during anteroposterior specification in Xenopus embryo. Dev Biol.

[48] Linne V, Stollewerk A (2011). Conserved and novel functions for Netrin in the formation of the axonal scaffold and glial sheath cells in spiders. Dev Biol.

[49] Lekven AC, Thorpe CJ, Waxman JS, Moon RT (2001). Zebrafish wnt8 encodes two wnt8 proteins on a bicistronic transcript and is required for mesoderm and neurectoderm patterning. Dev Cell.

[50] MacDonald BT, Tamai K, He X (2009). Wnt/beta-catenin signaling: components, mechanisms, and diseases. Dev Cell.

[51] MacDonald BT, He X (2012). Frizzled and LRP5/6 receptors for Wnt/β-catenin signaling. Cold Spring Harb Perspect Biol.

[52] Martí-Solans J, Godoy-Marín H, Diaz-Gracia M, Onuma TA, Nishida H, Albalat R, Cañestro C (2021). Massive gene loss and function shuffling in appendicularians stretch the boundaries of chordate wnt family evolution. Front Cell Dev Biol..

[53] Martin BL, Kimelman D (2009). Wnt signaling and the evolution of embryonic posterior development. Curr Biol.

[54] Martín-Durán JM, Hejnol A (2015). The study of Priapulus caudatus reveals conserved molecular patterning underlying different gut morphogenesis in the Ecdysozoa. BMC Biol.

[55] Mazo-Vargas A, Concha C, Livraghi L, Massardo D, Wallbank RWR, Zhang L, Papador JD, Martinez-Najera D, Jiggins CD, Kronforst MR, Breuker CJ, Reed RD, Patel NH, McMillan WO, Martin A (2017). Macroevolutionary shifts of WntA function potentiate butterfly wing-pattern diversity. Proc Natl Acad Sci USA.

[56] McClintock JM, Carlson R, Mann DM, Prince VE (2001). Consequences of Hox gene duplication in the vertebrates: an investigation of the zebrafish Hox paralogue group 1 genes. Development.

[57] McGregor AP, Hilbrant M, Pechmann M, Schwager EE, Prpic NM, Damen WG (2008). Cupiennius salei and *Achaearanea tepidariorum*: spider models for investigating evolution and development. BioEssays.

[58] McGregor AP, Pechmann M, Schwager EE, Feitosa NM, Kruck S, Aranda M, Damen WG (2008). Wnt8 is required for growth-zone establishment and development of opisthosomal segments in a spider. Curr Biol.

[59] McGregor AP, Pechmann M, Schwager EE, Damen WG (2009). An ancestral regulatory network for posterior development in arthropods. Commun Integr Biol.

[60] Mittmann B, Wolff C (2012). Embryonic development and staging of the cobweb spider Parasteatoda tepidariorum C. L. Koch, 1841 (syn.: *Achaearanea tepidariorum*; Araneomorphae; Theridiidae). Dev Genes Evol.

[61] Miyawaki K, Mito T, Sarashina I, Zhang H, Shinmyo Y, Ohuchi H, Noji S (2004). Involvement of Wingless/Armadillo signaling in the posterior sequential segmentation in the cricket, *Gryllus bimaculatus* (Orthoptera), as revealed by RNAi analysis. Mech Dev.

[62] Murat S, Hopfen C, McGregor AP (2010). The function and evolution of Wnt genes in arthropods. Arthropod Struct Dev.

[63] Nüsslein-Volhard C, Wieschaus E (1980). Mutations affecting segment number and polarity in Drosophila. Nature.

[64] Oda H, Akiyama-Oda Y (2020). The common house spider *Parasteatoda tepidariorum*. EvoDevo.

[65] O'Donnell BC, Jockusch EL (2010). The expression of wingless and Engrailed in developing embryos of the mayfly *Ephoron leukon* (Ephemeroptera: Polymitarcyidae). Dev Genes Evol.

[66] Panara V, Budd GE, Janssen R (2019). Phylogenetic analysis and embryonic expression of panarthropod Dmrt genes. Front Zool.

[67] Pechmann M, Prpic NM (2009). Appendage patterning in the South American bird spider *Acanthoscurria geniculata* (Araneae: Mygalomorphae). Dev Genes Evol.

[68] Pechmann M (2020). Embryonic development and secondary axis induction in the Brazilian white knee tarantula *Acanthoscurria geniculata*, C. L. Koch, 1841 (Araneae; Mygalomorphae; Theraphosidae). Dev Genes Evol.

[69] Perrimon N, Pitsouli C, Shilo BZ (2012). Signaling mechanisms controlling cell fate and embryonic patterning. Cold Spring Harb Perspect Biol.

[70] Pflugfelder GO, Eichinger F, Shen J (2017). T-Box genes in Drosophila limb development. Curr Top Dev Biol.

[71] Prpic NM, Janssen R, Wigand B, Klingler M, Damen WG (2003). Gene expression in spider appendages reveals reversal of exd/hth spatial specificity, altered leg gap gene dynamics, and suggests divergent distal morphogen signaling. Dev Biol.

[72] Prpic NM, Janssen R, Damen WG, Tautz D (2005). Evolution of dorsal-ventral axis formation in arthropod appendages: H15 and optomotor-blind/bifid-type T-box genes in the millipede *Glomeris marginata* (Myriapoda: Diplopoda). Evol Dev.

[73] Prpic NM, Schoppmeier M, Damen WG (2008). Collection and fixation of spider embryos. CSH Protoc..

[74] Prpic NM (2008). Parasegmental appendage allocation in annelids and arthropods and the homology of parapodia and arthropodia. Front Zool.

[75] Prühs R, Beermann A, Schröder R (2017). The roles of the Wnt-Antagonists Axin and Lrp4 during embryogenesis of the red flour beetle *Tribolium castaneum*. J Dev Biol.

[76] Quade FSC, Holtzheimer J, Frohn J, Töpperwien M, Salditt T, Prpic NM (2019). Formation and development of the male copulatory organ in the spider *Parasteatoda tepidariorum* involves a metamorphosis-like process. Sci Rep.

[77] Ring L, Neth P, Weber C, Steffens S, Faussner A (2014). β-Catenin-dependent pathway activation by both promiscuous “canonical” WNT3a-, and specific “noncanonical” WNT4- and WNT5a-FZD receptor combinations with strong differences in LRP5 and LRP6 dependency. Cell Signal.

[78] Routledge D, Scholpp S (2019). Mechanisms of intercellular Wnt transport. Development.

[79] Schönauer A, Paese CL, Hilbrant M, Leite DJ, Schwager EE, Feitosa NM, Eibner C, Damen WG, McGregor AP (2016). The Wnt and Delta-Notch signalling pathways interact to direct pair-rule gene expression via caudal during segment addition in the spider *Parasteatoda tepidariorum*. Development.

[80] Schwager EE, Schoppmeier M, Pechmann M, Damen WG (2007). Duplicated Hox genes in the spider *Cupiennius salei*. Front Zool.

[81] Schwager EE, Sharma PP, Clarke T, Leite DJ, Wierschin T, Pechmann M, Akiyama-Oda Y, Esposito L, Bechsgaard J, Bilde T, Buffry AD, Chao H, Dinh H, Doddapaneni H, Dugan S, Eibner C, Extavour CG, Funch P, Garb J, Gonzalez LB, Gonzalez VL, Griffiths-Jones S, Han Y, Hayashi C, Hilbrant M, Hughes DST, Janssen R, Lee SL, Maeso I, Murali SC, Muzny DM, Nunes da Fonseca R, Paese CLB, Qu J, Ronshaugen M, Schomburg C, Schönauer A, Stollewerk A, Torres-Oliva M, Turetzek N, Vanthournout B, Werren JH, Wolff C, Worley KC, Bucher G, Gibbs RA, Coddington J, Oda H, Stanke M, Ayoub NA, Prpic NM, Flot JF, Posnien N, Richards S, McGregor AP (2017). The house spider genome reveals an ancient whole-genome duplication during arachnid evolution. BMC Biol.

[82] Setton EVW, Sharma PP. A conserved role for arrow in posterior axis patterning across Arthropoda. Dev Biol. 2021 (S0012-1606(21)00039-7). 10.1016/j.ydbio.2021.02.006 33607111

[83] Sharma PP, Schwager EE, Extavour CG, Giribet G (2012). Hox gene expression in the harvestman *Phalangium opilio* reveals divergent patterning of the chelicerate opisthosoma. Evol Dev.

[84] Somorjai IML, Martí-Solans J, Diaz-Gracia M, Nishida H, Imai KS, Escrivà H, Cañestro C, Albalat R (2018). Wnt evolution and function shuffling in liberal and conservative chordate genomes. Genome Biol.

[85] Stollewerk A, Schoppmeier M, Damen WG (2003). Involvement of Notch and Delta genes in spider segmentation. Nature.

[86] Svendsen PC, Formaz-Preston A, Leal SM, Brook WJ (2009). The Tbx20 homologs midline and H15 specify ventral fate in the Drosophila melanogaster leg. Development.

[87] Svendsen PC, Phillips LA, Deshwar AR, Ryu JR, Najand N, Brook WJ (2019). The selector genes midline and H15 control ventral leg pattern by both inhibiting Dpp signaling and specifying ventral fate. Dev Biol.

[88] Swarup S, Verheyen EM (2012). Wnt/Wingless signaling in Drosophila. Cold Spring Harb Perspect Biol.

[89] Turetzek N, Prpic NM (2016). Observations on germ band development in the cellar spider *Pholcus phalangioides*. Dev Genes Evol.

[90] Turetzek N, Pechmann M, Schomburg C, Schneider J, Prpic NM (2016). Neofunctionalization of a duplicate dachshund gene underlies the evolution of a novel leg segment in arachnids. Mol Biol Evol.

[91] van Amerongen R, Nusse R (2009). Towards an integrated view of Wnt signaling in development. Development.

[92] Wiese KE, Nusse R, van Amerongen R (2018). Wnt signalling: conquering complexity. Development.

[93] Williams TA, Nagy LM (2017). Linking gene regulation to cell behaviors in the posterior growth zone of sequentially segmenting arthropods. Arthropod Struct Dev.

[94] Yan D, Lin X (2009). Shaping morphogen gradients by proteoglycans. Cold Spring Harb Perspect Biol.

