# Supplementary figures and images for: A chelicerate Wnt gene expression atlas: novel insights into the complexity of arthropod Wnt-patterning
Source: EvoDevo. 2021 Nov 9;12:12. doi: 10.1186/s13227-021-00182-1 (PMC8579682; doi:10.1186/s13227-021-00182-1)

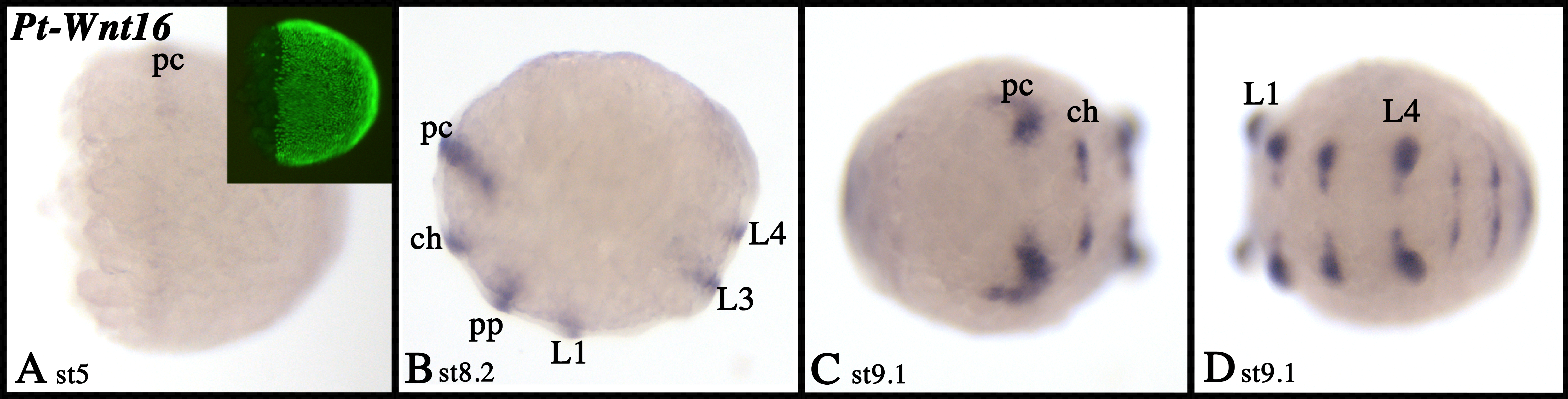

Supplement: Supplementary file 9 — Additional file 9: Fig. S9. Early expression of Parasteatoda Wnt16. In all panels, anterior is to the left, ventral views (except panel B (lateral view)). Inlay picture in A represents SYBR-green image of the embryo shown in the regular panel. Developmental stages are indicated. Abbreviations as in Fig. 4. [file 13227_2021_182_MOESM9_ESM.tif]
