# Supplementary material for: A chelicerate Wnt gene expression atlas: novel insights into the complexity of arthropod Wnt-patterning
Source: EvoDevo. 2021 Nov 9;12:12. doi: 10.1186/s13227-021-00182-1 (PMC8579682; doi:10.1186/s13227-021-00182-1)
Supplement: Supplementary file 12 — Additional file 12. Primers. [file 13227_2021_182_MOESM12_ESM.rtf]

Acanthoscurria geniculataWnt1.1_fw1		GTCACGCCGATAAATCTACCWnt1.1_fw2		GCTAAAGGAATGAAAACGGCWnt1.1_bw1		TCTTGCACGTCTTACACTCCWnt1.1_bw2		CCAGTGAAAAGTGCAGTTACWnt1.2_fw1		GATGTTCATCCCGCTTCTCAWnt1.2_fw2		CATATTACAGCAGCGAAAGGWnt1.2_bw1		CTACAGGCATGTGTGGATAGWnt1.2_bw2		TACAGTTGCACCGTTCCTGT		Wnt2_fw1		CGATGAGGATTATCAGACACWnt2_fw2		CCTTTTTATGCAGCCTCAAGWnt2_bw1		TTAGTTCTCCGTTGCTGCTTWnt2_bw2		TGAATGTCTTTCCACTCCCT	Wnt4.1_fw1		CACGGACTACAGATCGGATTWnt4.1_fw2		GATCCTCTTACCCTCTGGAAWnt4.1_bw1		TACTTCTACGTCGGTTTCACWnt4.1_bw2		CGTAGCAGCACCAGTAGAATWnt4.2_fw1		GTTCTGTAGAGAGCAGACGCWnt4.2_fw2		CGGAAAAACTGGAGCTTCGGWnt4.2_bw1		GCAGAAATGGCGTGGACGAAWnt4.2_bw2		GATTCCCTCGTTCCTTCTGAWnt5_fw1		GCGAATAGGGATCAACGAGTWnt5_fw2		GCAGTTCAGGCACAGGAGATWnt5_bw1		ACTGAATGTCCACCATGTAAAWnt5_bw2		GCACTTGCATCGTTCTTTGAT	Wnt6_fw1		ATCCTCCTAGTGATGCTGTGWnt6_fw2		ATCTACATGCTTCACGTCAGWnt6_bw1		GACAACGACTGTATGTTTGC Wnt6_bw2		CTGTCACAAGTATCACACTG	Wnt7.1_fw1		TGTTCCTGGTGATTTGCTTCWnt7.1_fw2		CCCTATGCTAATTTCAGAGCWnt7.1_bw1		GCATACTTTTCGGTCCTTTCWnt7.1_bw2		TTCTTTGCACTCTTCGCAGT	Wnt7.2_fw1		GGCAAAGAAGCCAGTGGTCCWnt7.2_fw2		CCAGTGGTCCCAACCTGCGAWnt7.2_bw1		GCTTGTGTCACTGCATATGTWnt7.2_bw2		TATGTCACTCCTGCTGACGTWnt11_fw1		ACAATCAAGCGGGAAGAAGGWnt11_fw2		ATGTCATGGAGTATCTGGGTWnt11_bw1		TCTTCTATCACGTTCATCACAWnt11_bw2		CTTTGTTCTTTTCCTACAGCAWnt16_fw1		TGTTAGTGTGGACCTCTTTCWnt16_fw2		CCTGGGCTAATTGGATGTACWnt16_bw1		CGTGTAGATTTCGTCCATAGWnt16_bw2		GTCTCACAAGTTTTACACCG	WntA_fw1		ATGCTCTTCACTCGAAGGGTWntA_fw2		TGTTTCTCGTAACTGTGCTGWntA_bw1		CAGTTGCAGTAATGTTCTTCWntA_bw2		CTTGAATCGACACATCTCGC	Parasteatoda tepidariorumWnt2_fw1		GAGTGCAAGAATAGCTGTTGWnt2_fw2		TTTGATGGCGTTCTGTTGTCWnt2_bw1		TGTCGTAACCCTTGCCACAAWnt2_bw2		CGAATCTCGGTCACATCTTCWnt4_fw1		ACAGCAAACCTTCTAGTTCCWnt4_fw2		CGAGAAGAAGACGATCACTGWnt4_bw1		CCAGTAGAACTTACAATCGCWnt4_bw2		TGCTTGTAAAACCTCGTCCAWnt5_fw1		AGACGTAAAGTGGTTGCAGAWnt5_fw2		CGATAGGAACATGGATGAACWnt5_bw1		TGTCTTGCACTCAACGTAACWnt5_bw2		GCACTTGCAGCGTTCTTTGAWnt6_fw1		GCGTCGTATGAGAGGGAAGCWnt6_fw2		GCTATCACTGAAGGATCGAAWnt6_bw1		CAGCTTCGTAACCTCTCCCAWnt6_bw2		ACGAGTGATTGCAGTGTCTTWnt7.2_fw1		ATCCCTGCTCACTGACTATCWnt7.2_fw2		GACAACGGCGGCTGCTTTTAWnt7.2_bw1		CACGAATACACTTCTGTCCTWnt7.2_bw2		CGACAATCCACGTAACAGCAWnt16_fw1 		GCCAGCGAACAATCCTCAATWnt16_fw2		TCAGTGCCAGGTCTTGTGATWnt16_bw1		GCATTTGACATAGCAACACCWnt16_bw2		GGCAGCGTTCTAATCTTCTGWntA_fw1		GCTTTCTGTTGCTCTTGCCTWntA_fw2		GCTGGTGGCTATTAGGAATGWntA_bw1		TTCACAATGGACTTTGCAGCWntA_bw2		AATCTGCAACCATCCATTCCWnt1			clone as used in Heingard et al. 2019Wnt7.1 			clone as used in Janssen et al. 2010Wnt8			clone as used in Janssen et al. 2010Wnt11.1			clone as used in Janssen et al. 2010Wnt11.2			clone as used in Janssen et al. 2010Phalangium opilioWnt1_fw1		TCGATCAGGGGAGGAAATAAWnt1_fw2		TTCATGGATATTAACGTGCCWnt1_bw1		CTTGCACTTTACTTGACAACWnt1_bw2		GCAATTACACCTTTCCCATTWnt4_fw			GCTTCCTTTGTTCACGCCATWnt4_bw			GCTTTCAACATCCCTATCGCWnt5_fw1		TTAAACGGCGATGGACTGTGWnt5_fw2		GCTACGTTCTCCTGCTACTTWnt5_bw			GTAGCTGGCGAGGTTTTGTTWnt6_fw1		ATCAGTTTCGGAACAGAAGGWnt6_fw2		CCGCTGATAAAAGGTCGCTTWnt6_bw1		CGTTCTTTTATCGTGGCAAGWnt6_bw2		CGGCAATTATAGGAGACGTTWnt7_fw			CGTCGAACCAAAGAGACCGAWnt7_bw			TATTATAATGAACATTTATTWnt8_fw1		GATTGGCGGTCAAGAAGACAATGGWnt8_fw2		CGCCACTAGAACTTGCCTGATGAAWnt8_bw1		TAATAAATAATGATTGTGAGGTTTWnt8_bw2		GAGGTTTAAAATTACACGTGATAAWnt9_fw1		AGCTTTGAGATTAGGGGTCAWnt9_fw2		TTGGTGTCGTAAACAGTTCGWnt9_bw1		CCGACGATCTCACTTGAACAWnt9_bw2		CCCTTCCGCAACACATAATCWnt10_fw1		TCGAGGGAGTTTTGGAAATGWnt10_fw2		TCTAACCACCACGGCAAATCWnt10_bw1		TGACCCATTCAACCGTGTTAWnt10_bw2		GCATTTGCGACTACGTTTTCWnt11_fw1		TGCAACGATAACCTCGAGTGGGWnt11_fw2		GGTCAACAGGCACAACTACAAAGCWnt11_bw1		CANGTYTTRCAYTTNACRTARCARCA (CCYVKCKTC)Wnt16_fw1		CCTACGCAACTTTAACACTCWnt16_fw2		GTATTTAGGTATGGTTGGCGWnt16_bw1		CACCAATGAAACTTGCAGTGWnt16_bw2		TATTATAGCCACGTCCGCAAWntA_fw1		ATCGACGAGTGCCAGTTTCAGTTTWntA_fw2		ACTTTCAACAACAGTTCGTCCGTCWntA_bw			CRTGRCAYTTRCANACNARYTCCAT (MELVCKCHG)Pholcus phalangioidesWnt1_fw			GTAGTGGGTGGAGGCGTGGTTTTTAWnt1_bw			GTATCCCCTCCCGCAACATAACAGGWnt2_fw			CGGCGTAGAAATCCCAGTGTGATGAWnt2_bw			CTACTGGTGTGAACGGACCCTACCTWnt4_fw			CTTGGTCAGTCGTCTATCCCGGAGAWnt4_bw			CGGACTTAGTTCAACGATGGGATGCTWnt5_fw			TTCTGTTCGGAGGATGCAAGTCGACWnt5_bw			GCGTCCGAGTGTTCTTGTCTATCCCWnt6_fw			TATTCCCTACGTCACGCAAAACCCGWnt6_bw			TCGAAGTGTGGTTGCATTCCCTACCWnt7.1_fw		ACGGGTGTGTGACAATAAGTGCCATWnt7.1_bw		TGTTGTGCAGGTTCATGAGCGATCTWnt7.2_fw		GCCTAAATGCTGCTGACTTTGCTCGWnt7.2_bw		GCAAGTGTACTCCTCTGTCCTTTCGGWnt8_fw			GCAGGGATTGAATTTCACCAGCACAWnt8_bw			TTCCCCATCACGTTTCGAACACTGTWnt11.1_fw		CCTCTTGTTTTCGACGACCTCTGCTWnt11.1_bw		GACAACGTCAAGTACGGAGTCCGGWnt11.2_fw		GTCCGTCCAAAAAGCAAACCATGGGWnt11.2_bw		CAGGGGGTCGGTAGGCAAATTCTTTWnt16_fw		TTCTTTGATGTTGTGCGTGCGTGTGWnt16_bw		AGGTAATTGGGGAGGGATGTCGATTGWntA_fw			GGATCACACCTGAGGATGCGATACGWntA_bw			TGATTGGGAGATGATGAACCGGCAC
